# Effect of Meat Price on Race and Gender Disparities in Obesity, Mortality and Quality of Life in the US: A Model-Based Analysis

**DOI:** 10.1371/journal.pone.0168710

**Published:** 2017-01-03

**Authors:** Allison Pitt, Eran Bendavid

**Affiliations:** 1 Department of Management Science & Engineering, Stanford University, Stanford, California, United States of America; 2 Division of General Medical Disciplines, Stanford University, Stanford, California, United States of America; 3 Center for Health Policy and the Center for Primary Care and Outcomes Research, Stanford University, Stanford, California, United States of America; Old Dominion University, UNITED STATES

## Abstract

**Introduction:**

There are large differences in the burden and health implications of obesity by race and gender in the US. It is unclear to what extent policies modifying caloric consumption change the distribution of the burden of obesity and related health outcomes. Meat is a large component of the American diet. We investigate how changing meat prices (that may result from policies or from exogenous factors that reduce supply) might impact the burden of obesity by race and gender.

**Methods:**

We construct a microsimulation model that evaluates the 15-year body-mass index (BMI) and mortality impact of changes in meat price (5, 10, 25, and 50% increase) in the US adult population stratified by age, gender, race, and BMI.

**Results:**

Under each price change evaluated, relative to the status quo, white males, black males, and black females are expected to realize more dramatic reduction in 2030 obesity prevalence than white females. Life expectancy gains are also projected to differ by subpopulation, with black males far less likely to benefit from an increase in meat prices than other groups.

**Conclusions:**

Changing meat prices has considerable potential to affect population health differently by race and gender. In designing interventions that alter the price of foods to consumers, it is not sufficient to assess health effects based solely on the population as a whole, since differential effects across subpopulations may be substantial.

## Introduction

More than one-third of US adults are obese.[1] Yet, different populations are affected by the obesity epidemic to varying extents: the prevalence of obesity is 33% among white women, but nearly 60% among black women.[2] These differences in the distributions of BMI contribute to the four-year disparity in life expectancy between whites and blacks.[3] Obesity leads to increased mortality through higher rates of diseases, including heart disease, diabetes, stroke, and obesity-related cancers.[4] Health outcomes for white individuals with these conditions tend to be better than those for blacks in part due to greater access to higher quality healthcare.[5–7] Obesity also results in increased healthcare costs, and is estimated to raise individual annual medical costs by $3,300 (2015 dollars).[8,9] The health sequelae of obesity, however, vary based on race and gender: black women on the lower end of overweight (25.0–27.4 kg/m^2^ BMI) do not have a statistically higher risk of mortality than those on the upper end of the normal range (22.5–24.9 kg/m^2^ BMI), but the same is not true for white women, black men, or white men. [10] The heterogeneity by race and gender of the burden and health implications of obesity suggest that untargeted efforts to change obesity—such as through upstream policies like beverage taxation—may have complex and unintuitive implications for the distribution and health impacts of obesity.[11]

Policies to raise the price of some foods and thus encourage reduced calorie consumption are commonly applied without considerations for the differential downstream impacts by race and gender. Such policies range from increasing price to consumers (e.g., “fat taxes”) to placing restrictions on sellers (e.g., the New York City “soda ban”). In addition to policy interventions, exogenous factors (e.g., climate change or biofuel policies) may cause long-term change in food price. Change in food price may influence consumption behavior and ultimately shape obesity and public health.

This study focuses specifically on the consumption of meat in the US. Meat comprises a substantial portion of individuals’ diets, is generally the most expensive type of calorie, and its price may be subject to large changes. Additionally, US prices for meat may be particularly sensitive to growing demand for meat in the developing world.[12] While in this study we assess the impact of change in the price of meats, an analogous study could be performed for any type of food.

There have been numerous studies investigating the potential obesity impact of price changes for specific foods.[13] However, to our knowledge, no studies have modeled the effect of meat price on the prevalence and distribution of obesity in adults. Sturm and Datar empirically analyze the association between food prices and changes in body mass index (BMI) among elementary school children.[14] We, however, draw our attention to the adult population—a population directly in control of their own consumption, and thus more likely to respond to price changes.

## Methods

### Simulated Population

We simulated a population of 100,000 individuals resembling the 2013 US adult population by gender, race, and age.[15] At baseline each individual was characterized by total caloric consumption, inferred resting metabolic rate (estimated by their weight, height, age, and gender[16]) and physical activity level (the ratio of total energy expenditure to resting metabolic rate). Fig A in S1 Appendix shows the distribution of BMIs (15.0–18.4 kg/m^2^ [underweight], 18.5–24.9 kg/m^2^ [normal weight], 25.0–29.9 kg/m^2^ [overweight], 30.0–60.0 kg/m^2^ [obese]) by gender and race. Within each race-gender group, we determined the proportion of calories contributed by each food group based on nationally representative 24-hour dietary recall data.[17] We used this to determine initial total calories from meats —red meat, white meat, and seafood. Table 1 highlights key model parameters.

**Table 1:** Selected Model Parameters.

	Subpopulation				Reference
	White males	Black males	White females	Black females	
**Initial BMI distribution**					(10)
15.0–18.4 kg/m^2^	3%	4%	4%	3%	
18.5–24.9 kg/m^2^	24%	28%	35%	17%	
25.0–29.9 kg/m^2^	38%	30%	27%	26%	
30.0–60.0 kg/m^2^	35%	39%	34%	53%	
**Proportion of calories from meats (95% CI)**	17.8% (15.8–19.8)	22.9% (20.4–25.3)	15.3% (13.3–17.3)	21.5% (19.2–23.8)	(12)
**Utility value**					(18)
15.0–24.9 kg/m^2^	0.9	0.88	0.88	0.85	
25.0–29.9 kg/m^2^	0.87	0.89	0.87	0.85	
30.0–60.0 kg/m^2^	0.85	0.83	0.83	0.74	
**Relative risk for mortality**					(4)
15.0–18.4 kg/m^2^	1.37	1.58	1.42	1.52	
18.5–19.9 kg/m^2^	1.31	1.58	1.10	1.21	
20.0–22.4 kg/m^2^	1.12	1.14	1.00	1.07	
22.5–24.9 kg/m^2^	1.00	1.00	1.00	1.00	
25.0–27.4 kg/m^2^	1.01	1.02	1.07	1.09	
27.5–29.9 kg/m^2^	1.11	1.03	1.16	1.19	
30.0–34.9 kg/m^2^	1.30	1.18	1.30	1.27	
35.0–39.9 kg/m^2^	1.63	1.31	1.60	1.49	
40.0–60.0 kg/m^2^	1.92	1.31	2.06	1.69	
**Price elasticity of demand**					(13)
Red meat	-0.52	-0.52	-0.52	-0.52	
White meat	-0.46	-0.46	-0.46	-0.46	
Seafood	-0.53	-0.53	-0.53	-0.53	

### Effect of Price Change on Consumption

A one-time, permanent change in the price of red meat, white meat, and seafood (uniformly) is simulated, causing individuals to shift their food consumption in accordance with the price elasticities of demand for meats. Price elasticity of demand relates price changes to changes in quantity of a good purchased: if the price of a good increases by X% and it results in a Y% reduction in quantity consumed, the elasticity of demand for that good is –Y/X. In illustration: if a good has -0.5 price elasticity of demand, a 10% price increase for that good would result in a 5% reduction in quantity consumed.

When the price of one good increases, consumers tend to reduce consumption of that good and may change their consumption of other goods. Cross-price elasticity estimates the change in consumption of a good in response to price change of another good. (For example, if the price of Pepsi Cola increased, people may buy more of its substitute, Coca Cola. Conversely, if the price of peanut butter increased, people may buy less of its compliment, jelly.) Data on consumer behavior in the face of food price change suggests that most food groups serve as complements to meats (i.e., an increase in meat price results in a reduction in other food consumption in addition to meat).[18] Our base case assumes no complementarity given the scant empirical evidence, and we test this assumption in sensitivity analysis (Fig X and Y in S1 Appendix).

We determined each individual’s change in overall caloric consumption by estimating change in demand for meats given their original meat consumption level, elasticity of demand for meats, and the change in meat price. The change in caloric intake from meats gives their total change in consumption.

### Time Dynamics of Consumption and Weight Change

Historically, there have been nearly linear increases in average BMI and obesity prevalence over many years.[17] This is the result of an energy imbalance: in any given year, individuals consume more daily calories than they burn, which causes weight gain. The next year, individuals consume still more calories, causing additional weight gain. In our base case, we assume the tendency to consume more calories each year will continue. This gradual increase in food consumption, combined with the one-time change in consumption due to meat price change, yields individuals’ net change in consumption over time.

We evaluated the 15-year impact of a shift in individuals’ total caloric consumption on their bodyweight using equations validated by the National Institutes of Health.[19] These equations account for individuals’ current weight, resting metabolic rate (based on age, gender, height, and weight), level of physical activity, and change in daily caloric intake to determine expected weight at a later point in time. We believe 15 years is sufficiently long to see effects of change in consumption on weight, while not so long as to expect substantial shifts in parameters used to instantiate the model.

### Mortality

Annual risk of death for simulated individuals is based on gender, race, and age-specific mortality rates determined by the Centers for Disease Control,[3] which we adjusted for differential risk of death dependent on BMI.[10] Extreme BMIs—high and low—are associated with higher risk of death (Fig B in S1 Appendix), though this effect may be confounded by smoking and concurrent illness such as cancer.[10] Our base case assumes the J-shaped relationship estimated in the literature, and we test this assumption in sensitivity analysis (Fig G through K and U through W in S1 Appendix).

### Quality of Life

Because obesity is associated with a variety of comorbidities, decline in physical functioning, and increased pain,[20] we adjusted life years lived to account for decrement in quality of life using EQ-5D utility values. As shown in Table 1, BMI affects quality of life within each race-gender group differently.[21]

### Sensitivity Analysis

We tested the model’s sensitivity to many parameters and assumptions, including proportion of diet comprised of meat, elasticity of demand for meats, effect of food substitutes and complements, tendency to gradually increase consumption, impact of BMI on mortality risk, explicit modeling of smoking status, prevalence of disease in the population, and direction of meat price change (i.e., decrease). The analytic code is available from the authors upon request; all analyses were performed using R (CRAN).

## Results

### Model Calibration

**Fig 1:**
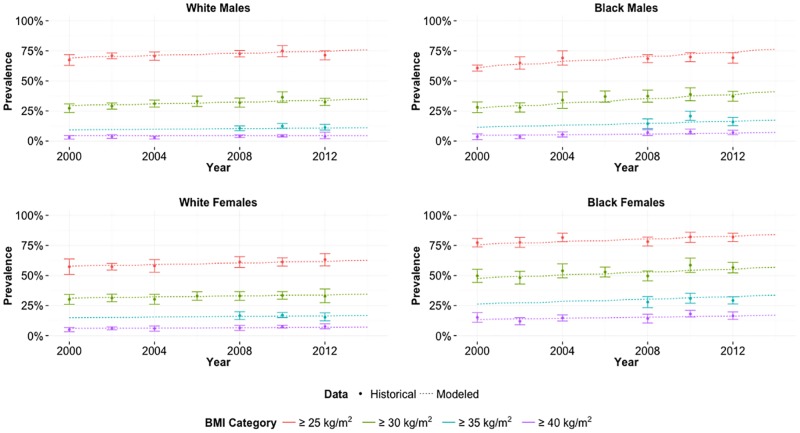
Model Calibration. Calibrated year-over-year increase in daily calories for each race-gender such that model predictions best fit proportion of subpopulation in each BMI range reported in literature.

We calibrated the model to observed historical trends in obesity rates from 1999 to 2012 for each subpopulation, assuming no change in meat price.[2,17,22–25] We assumed that every year individuals in each race-gender subpopulation consume some fixed number of additional daily calories, as determined by that necessary to best fit the prevalence of BMIs greater than 25, 30, 35, and 40 kg/m^2^ over time according to mean squared error. Table 2 indicates the additional daily calories eaten year over year. Fig 1 shows the model’s success in fitting the 95% confidence interval for each historical data point.

**Table 2:** Modeled Yearly Caloric Consumption Increases.

	Subpopulation			
	White Males	Black Males	White Females	Black Females
Yearly increase in daily calories (kcal)	5.7	12.3	4.7	8.7

### Base-Case Analyses

We simulated the effect a 0% (status quo), 5%, 10%, 25%, or 50% increase in meat price and observe the impact over 15 years on obesity prevalence, life years lived, and quality adjusted life years within each race-gender subpopulation.

#### Obesity prevalence

**Fig 2:**
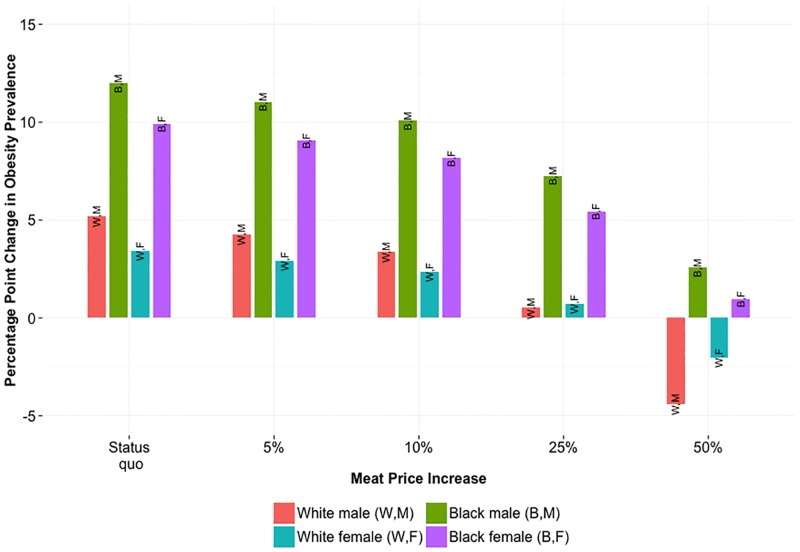
Change in Obesity Prevalence Over 15 Years Relative to Current Prevalence. Change in obesity prevalence by race-gender from 2015 to 2030 under each meat price increase.

Only for extreme increases in meat price greater than 25% did we see reduction in 2030 obesity prevalence relative to 2015 prevalence (Fig 2), all else being equal. Furthermore, blacks saw greater increases in obesity prevalence over 15 years than whites—due largely to their more rapid increase in caloric intake over time—such that even a 50% increase in meat price did not lower obesity relative to current levels.

**Fig 3:**
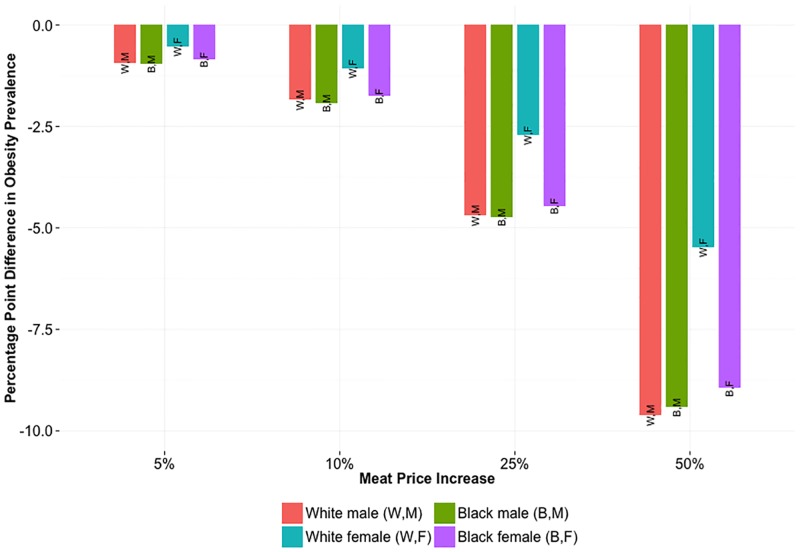
Difference in Obesity Prevalence after 15 Years Relative to Status Quo. Difference in 2030 obesity prevalence under specified meat price increase, relative to 2030 prevalence under no price change.

Fig 3 highlights the effect of an increase in meat price on 2030 obesity prevalence, relative to the projected 2030 prevalence under no price increase (status quo). The effect was consistent across white males, black males, and black females. The effect for white females was approximately half as dramatic, since their initial obesity prevalence and meat intake were relatively low. Fig D through E in S1 Appendix detail prevalence of obesity, overweight, and underweight over the 15-year time horizon for each group.

#### Life years

**Fig 4:**
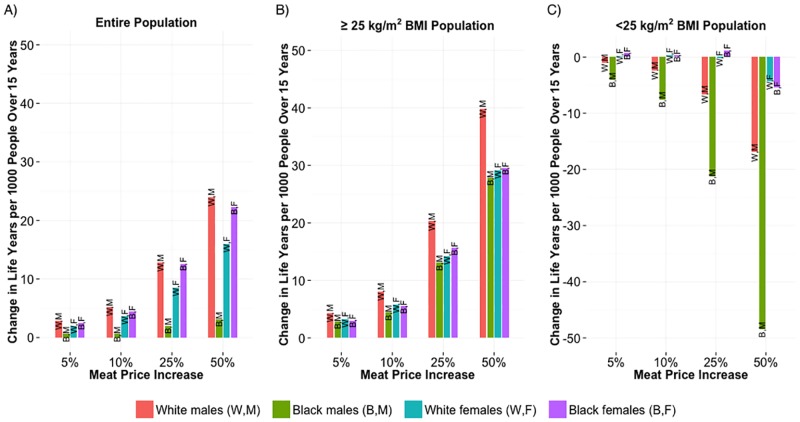
Difference in Life Years Lived Over 15 Years Relative to Status Quo. (A) Difference in life years lived over 15 years per 1000 people under specified meat price increase, relative to that under no price change. (B) Same as A, including only individuals with initial BMI greater than or equal to 25 kg/m2. (C) Same as A, including only individuals with initial BMI less than 25 kg/m^2^.

Increased meat price resulted in increased life years lived relative to the status quo (Fig 4A). White males and black females received the greatest mortality benefit relative to the status quo, while black males experienced the least benefit. Further dividing each race-gender group into individuals who had initial BMIs of at least 25 kg/m^2^ (overweight) versus less than 25 kg/m^2^ yielded additional insights. Those initially overweight experienced reduced mortality with increasing meat price (Fig 4B). (Overweight white males benefited the most as they avoided relatively high mortality risk at elevated BMIs.) However, among those not initially overweight, there was no mortality benefit with increasing meat price (Fig 4C).

The disproportionately small health benefit to black males following increased meat price resulted from shifting a substantial portion of that population into low-BMI, high-mortality zones without a large enough offsetting effect for high-BMI black males. (Black males have higher risk of mortality at low BMIs relative to the other groups, while their risk of mortality at high BMIs is lower than that for others.)

#### Quality of life

Quality adjusted life years (QALYs) lived relative to the status quo increased with increasing meat price (Fig F in S1 Appendix). Black females enjoyed the greatest gains in QALYs relative to the status quo in large part due to avoiding the hefty utility decrement associated specifically with obesity in black women.

### Sensitivity Analysis

Our sensitivity analyses highlight the drivers of the variability in health effects by race and gender following meat price changes. (Fig G through GG in S1 Appendix illustrate the findings from our sensitivity analyses.) Variability of the J-shaped BMI-mortality relationship is influential for our findings on the life expectancy of black males. If the impact of the BMI-mortality relationship for blacks were equal to that for whites, blacks males and females would experience a substantially greater mortality benefit from meat price increase (Fig G in S1 Appendix). The initial distribution of BMIs for each subpopulation was also influential. If the BMI distribution for blacks were equal to that for whites, increases in meat price would yield substantial mortality benefit for black males (Fig M in S1 Appendix).

## Discussion

We present an analysis of the variation in health effects by race and gender following a hypothetical shift in the price of meat in the US. We show that the variability in impact on obesity prevalence and life expectancy by race and gender is expected to be substantial. Price increases are expected to have a far smaller impact on obesity prevalence among white females over a 15-year period relative to the other subpopulations. We also show that life expectancy benefits from obesity reductions may not be realized by black males as a group, while white males and black females benefit the most. Additionally, at the present rate of growing caloric consumption, only very large increases in meat prices may be enough to lower obesity prevalence below current levels (and only among some groups), which suggests a limited health effect for policies such as taxes that target prices to reduce obesity.

Key drivers of the differential impact on subpopulations include (i) the non-monotonic relationship between obesity and mortality, such that both extremes of the BMI distribution pose elevated mortality risk, (ii) differing BMI distributions among different groups, and (iii) variation in food consumption patterns. With respect to the first driver, though there is heightened mortality risk for both high and low BMIs, the risk differs by race. Blacks are more adversely affected at low BMIs, while whites are more adversely impacted at high BMIs. The decrease in meat price causes high-BMI whites to benefit more than high-BMI blacks and low-BMI blacks to be harmed more than low-BMI whites. The difference in BMI distribution between groups is important because individuals on either end of the BMI spectrum are most impacted by a change in meat price. Differences between race-gender groups in the proportion of people in high- and low-BMI ranges means that some are more affected (benefited or harmed) by a change in meat price than others. Lastly, differences in meat consumption impact the degree to which groups are affected by a change in meat price, since those who tended to eat less meat prior to the price change incur less of a shock to their diet than those who depended more on meat.

The extent to which obesity prevalence is expected to decline relative to the present level following a price increase is driven by the degree of gradual consumption increase over time, since this trend determines the background secular increases in obesity, on which the effect of price increases is superposed. There is uncertainty about whether increasing consumption will continue at its historically observed level. In the event that this effect is reduced, we find that black males would experience a *reduction* in life expectancy at higher price increases, as the counterbalance against the harm from large price increases experienced by the underweight is diminished.

Our study addresses an important and complex question with the best available epidemiological data and methods. A randomized trial to project the distributional impacts of a price hike is clearly infeasible, so we bring together existing data from a variety of respected sources in a model to inform that question. Furthermore, we perform extensive sensitivity analyses to test the assumptions made in developing the model and understand the impact of parameters for which there is substantial uncertainty.

Our analysis has limitations. The model projects change in total calories in response to a change in meat prices to predict the resulting change in BMI and mortality risk. However, the model predictions assume that increasing meat prices would lead to a reduction in total calories consumed. There is evidence to suggest that the calorie reductions modeled are conservative, yet there remains substantial uncertainty around how individuals’ consumption patterns would change in the long term with a sustained shift in meat price. Furthermore, if individuals alter their macronutrient consumption breakdown—not explicitly modeled in this analysis—this has potential for unexpected health effects beyond that which can be predicted according to total caloric consumption.

Additionally, the projected differential mortality effects across subpopulations following a change in meat prices results from a reported differential BMI-mortality risk by race and gender. The concerns over confounders of the BMI-mortality relationship mean that our base estimates are biased if the same confounders are distributed differentially by race and gender.

Finally, our base case model is calibrated to historical trends of gradually increasing calorie consumption over time. However, the extent to which this 30-year trend will persist is unknown, and there is some early evidence of calorie intake plateauing in some populations.[26] If we relax this assumption, we find that meat price increases may not need to be as dramatic as suggested by this analysis to reduce obesity rates relative to the present over the next 15 years.

## Conclusions

The finding that the differential effect across subpopulations is large, and that black males appear more susceptible to adverse health effects from an increase in the price of meat has notable policy implications. Firstly, in a heterogeneous population, examining the mean population benefits of a policy or intervention may fail to identify variability in benefits, or even harms, in subpopulations. That is, upstream policies with mean population health benefits may cause harm and exacerbate pre-existing disparities. In the context of our study, we found that price increases that improved longevity and quality of life for most of the population were also consistently related to reduced life expectancy among black males. This highlights the difficulty in designing untargeted interventions that will benefit all segments of the population roughly equally. Additionally, interventions designed to improve health by modifying food prices may prove particularly regressive, due to the difficulty of targeting price changes to specific groups.

Our model highlights the considerable potential of changes in meat prices to have divergent effects on the health of different populations. While we focus this study on the price of meat, given the relationship between meat price and the price of feed for livestock, our findings also have implications for other phenomena that affect the price of corn such as price speculation and federal farm subsidies. Moreover, our insights have broad applicability to policy development, as examining the variability of health effects will be important when designing upstream policy interventions in general.

## Supporting Information

S1 Appendix The appendix contains supplemental information about the methods and results, including several additional figures (S1 Fig A through GG). (PDF)
