# Supplementary material for: Effect of Meat Price on Race and Gender Disparities in Obesity, Mortality and Quality of Life in the US: A Model-Based Analysis
Source: PLoS One. 2017 Jan 3;12(1):e0168710. doi: 10.1371/journal.pone.0168710 (PMC5207744; doi:10.1371/journal.pone.0168710)
Supplement: S1 Appendix — (PDF) [file pone.0168710.s001.pdf]

Supplemental Methods

Microsimulation Model

Our simulated population of 100,000 individuals was stratified by age (18 to 100), gender, and race (non-Hispanic white and non-Hispanic black), in proportion to the demographic characteristics of the current U.S. adult population, with 18 year-olds maturing into the adult population each year. Specifically, the proportion of non-Hispanic white males, non-Hispanic white females, non-Hispanic black males, and non-Hispanic black females in several age groups (18-29, 30-39, 40-49, 50-59, 60-69, 70-79, 80 and older) match that of the U.S. The simulated model duration is 15 years. Each year, individuals may experience a change in weight and have a chance of dying. We run 10 trials of our simulation, each with a different, randomly generated population and assess the average results.

Simulated Population

The initial simulated BMI for each individual is drawn from a BMI distribution of the corresponding age-race-gender population in the 2013 National Health and Nutrition Examination Survey (NHANES).[1] The distributions of BMIs were matched according to 5th, 10th, 15th, 25th, 50th, 75th, 85th, 90th, and 95th percentiles and bounded by 15 kg/m^2 and 60 kg/m^2. Fig A depicts the initial distribution of BMIs by subpopulation. Individual heights were assigned according to mean and standard deviation reported from NHANES. Weights were computed given BMI and height.

Individuals were permitted to mature into the population or leave the population through death. For simplicity, we assumed a birth rate consistent with approximately zero

population growth (equal to the death rates[2]) and did not allow the birth rate to vary over the course of the simulation with the size of the model population.

Baseline Food Consumption

Average consumption patterns (proportional contribution of each food group to the total energy intake) for each race-gender were identified according to NHANES food recall data, adjusting for survey weighting. Dietary recall is biased toward under-reporting consumption, which makes it a poor tool for assessing total energy intake. Furthermore, this trend toward under-reporting tends to be more pronounced among women than men.[3] In light of these biases, we did not to use dietary recall data to estimate total caloric consumption, and instead use it to estimate relative consumption across the various food groups. We believe that the likelihood of bias in relative consumption across food groups would be diminished and unlikely to vary notably by demographic.

Foods were classified into one of the following groups: red meat, white meat, seafood, grain, dairy, eggs, fruit, vegetables, legumes/nuts/seeds, fats, sweets, sugar-sweetened beverages. Meats that could not be classified as red meat, white meat, or seafood were assumed to have the same breakdown (red meat/white meat/seafood) as meats that could be readily classified.

Effect of Price Change on Consumption

Price elasticities of demand and cross price elasticities were drawn from the study performed by Basu et al.,[4] in which elasticities were determined according to the

quadratic almost ideal demand system[5], combining NHANES consumption data with USDA price history data. This source was selected due to the comprehensive inclusion of own- and cross-price elasticity estimates for all food groups represented in the NHANES 24-hour recall data. There are many other sources for data on food price elasticity, though they tend to be less comprehensive. Andreyeva et al. performed a systematic review of own-price elasticity of demand for several foods. The own-price elasticity values for white meat and red meat estimated by this review were somewhat higher than those used in our base case, but well within the range explored in our sensitivity analysis.[6] We acknowledge that elasticity estimates are accurate within some suitably small range of price changes, and that for larger price changes, predictions for change in consumption, may be inaccurate.

Time Dynamics of Consumption and Weight Change

The time scale over which weight change occurs in response to a change in consumption is long. Within the first year following a shift in consumption, approximately 50% of the weight change towards equilibrium occurs, however it takes approximately three years to get 95% of the way to a new equilibrium weight.[7]

We modeled the combined effect of change in consumption due to the price change and change in consumption due to the exogenous tendency to gradually increase consumption as additive. (The impact of the former is only directly observed over approximately the first three years of the simulation, since it is a one-time change.) While it is conceivable that the tendency to gradually increase one's consumption may be affected

by a shift in price, we assumed that this is not the case. However, in our sensitivity analysis we test different assumptions about the tendency to increase consumption over time.

The equations we used to determine weight in response to a change in caloric intake assume that current weight is at equilibrium prior to the change in intake. While this constraint is amenable to a one-time shift in consumption, it presents a challenge for assessing the impact of multiple changes in intake over relatively short period of time (e.g., tendency for gradual increase in intake). Therefore, we choose to consider the tendency for individuals to gradually increase their intake over time not on a yearly basis, but instead assumed these gradual increases take place every three years (and that the increase in consumption is three times as large as it would have been if increased yearly). (The wavy nature of the prevalence curves in Fig C through E is an artifact of the three-year time span over which we model gradual change in consumption in order to allow weight to equilibrate before assessing the impact of another small change in consumption.)

The equation relating change in consumption to weight change accounted for individuals' physical activity level. For simplicity, all members of the population were assumed to have the same physical activity level: a ratio of 1.5 total energy expenditure to resting metabolic rate. However, evidence suggests that some groups are more physically active than others.[8] Specifically, men tend to be more physically active than women and younger adults tend to be more active than older adults, but difference between races is far less notable.

Mortality

BMI-dependent risk of death, given gender, race, smoker status, and disease status was based on data from the American Cancer Society's Cancer Prevention Study II.[9] For each race-gender subpopulation, we determined mortality risk ratios according to BMI by weighting the risk ratios given smoker and disease status according to estimated smoking and disease prevalence for the group as reported in the American Cancer Society's Cancer Prevention Study II at baseline. Unlike in the case of the other race-gender groups, the number of black males with BMIs greater or equal to 40 kg/m^2 in this study was too small to warrant assessment of their risk ratio for mortality, so risk ratio for mortality was assessed for black males greater than or equal to 35 kg/m^2 as opposed to separately for 35.0-39.9 kg/m^2 and greater than or equal to 40.0 kg/m^2. For each race-gender group, given the proportion of the population in each BMI category, and the mortality risk ratios for each BMI category, we used the CDC's overall mortality rate for the group to derive the mortality rates by race, gender, and BMI range (shown in Fig B).

There is considerable debate about whether the relationship between extreme BMIs (particularly low BMIs) and heightened mortality rates is causal.[9–15] Illness and smoking status are potential confounders. We performed sensitivity analysis to test the impact of explicitly accounting for differing risk of mortality for smokers versus nonsmokers by assigning smoker status and using differing risk ratios for mortality as identified by Patel.[9] We also evaluated sensitivity to the prevalence of illness in the population by testing the extremes: a population that has no disease, and a population in which every individual suffers from disease, applying the differing risk ratios for mortality associated with each.

Supplemental Results

Base-Case Analyses

Obesity Prevalence

An increase in the price of meat reduced obesity prevalence (BMI ≥ 30 kg/m^2) relative to the status quo in each race-gender group, as shown in Fig C. During the first three years, weight for individuals (and thus the trajectory of obesity prevalence) accounts for reduction in consumption in response to the price increase combined with the tendency to gradually increase consumption. The degree to which a given price increase impacts prevalence over this period depends on the extent to which the decrease in consumption corresponding to the price increase (which varies with the proportion of a group's calories derived from meat) is counterbalanced by the tendency to gradually increase consumption over this period. Following this initial period, the change in obesity prevalence rises steadily in accordance with the rate of gradual increase in caloric intake (which also varies by race-gender group). Overweight prevalence (BMI ≥ 25 kg/m^2) over 15 years, as depicted in Fig D, exhibits trends between subpopulations analogous to that for obesity. Trends in underweight prevalence (BMI < 18.5 kg/m^2) vary by subpopulation, as shown in Fig E.

Quality of Life

In addition to comparing QALYs lived relative to the status quo over all members of each gender-race group (Fig F1), we assessed this on the basis of initial BMI, as shown in

Fig F2 and Fig F3. Among the initially overweight, all race-gender groups experienced an increase in QALYs with increasing price. While black males who were not initially overweight suffered with respect to QALYs, non-overweight white males still received a QALY benefit from increasing meat price. Though these white males were not initially overweight, many of them were on the verge of becoming overweight, so the tendency to gradually increase caloric intake resulted in many of them becoming overweight. Thus the meat price increase posed a barrier to them becoming overweight and enabled them to enjoy the higher utility associated with being of normal weight.

Sensitivity Analysis

Impact of BMI on Risk Ratio for Mortality (Fig G through K)

There is significant uncertainty around the impact that BMI has on risk of mortality—especially for blacks, given their small samples sizes in the study from which this data is drawn. For this reason, we perform the following several sensitivity analyses. (Note that these what-if analyses around the impact of BMI on mortality would not impact obesity prevalence, so we present only the effects on life years lived.)

I. Assume black risk ratios of mortality by BMI are equal to that for whites (Fig G)

II. Reduction in black male risk ratios of mortality for BMI in 15-18.5 kg/m^2 and 18.5-20 kg/m^2 ranges to that of the 20-22.5 kg/m^2 BMI range (Fig H)

III. Reduction in black male risk ratios of mortality for BMI in 18.5-20 kg/m^2 range to that of the 20-22.5 kg/m^2 BMI range (Fig I)

IV. Assume black male risk ratio of mortality for BMI greater than or equal to 40 kg/m^2 is equal to that for whites (Fig J)

In each of these analyses, we find that black males, who in the base case received by far the least mortality benefit, are projected to enjoy substantial increases in life years with increasing meat price, relative to the base case. In analyses III and IV, black males are projected to achieve mortality benefit comparable to that of white women. In analysis I, both black males and black females benefit to an even greater extent than white males.

Additionally, in the base case, the relationship between BMI and risk of mortality is J-shaped, where both low and high BMIs indicate worsened health and increased chance of death. Although low BMIs are frequently indicative of illness, being underweight does not typically cause these illnesses—rather the illness causes the weight loss. To reduce potential for confounding, we investigate the impact of assuming no increase in risk of mortality for low BMI individuals (for all race-gender groups) by assigning a mortality risk ratio of 1.0 for BMIs below 25 kg/m^2 (Fig K). Life years lived across all subpopulations are increased, with black males' life years increasing dramatically such that their relative mortality benefit is comparable to that for white women.

Initial BMI Distributions (Fig L and M)

If the initial distribution of BMIs among blacks were equal to that for whites, with respect to gender, 15-year-out obesity prevalence under various price increases relative to no price change is reduced more dramatically for black males and less dramatically for black females, compared to the base case in which the BMI distributions vary by both race

and gender. The reason for this is the proportion of white males who are overweight or have obesity is greater than the corresponding proportion of black males, so if the black BMI distribution were equal to that for whites, the increase in meat price (and resulting decrease in consumption) has greater potential to drive obesity prevalence lower. Conversely, for black females, if their BMI distribution matched that for white females (who have lower prevalence of overweight and obesity), there is less potential for an increase in meat price to drive obesity prevalence lower.

The impact on life years, in this scenario in which black BMI distributions match that for whites, is that black males benefit more from a meat price increase than in the base case, and black females benefit less. Benefit to black females is reduced because far fewer of them (than in the base case) are overweight or have obesity, so there's less potential to benefit from decreasing caloric intake, while more of them have low BMIs and increased risk of harm from a meat price increase. Conversely, benefit to black males increases because in this what-if analysis, fewer of them have low BMIs, and are thus not as subject to harm in response to a meat price increase.

Food Consumption Patterns (Fig N and O)

We assess how meat price increase affects obesity prevalence and life years lived under the scenario in which the proportion of black individuals' diets comprised of meat matches that of whites, with respect to gender. This represents a decrease in the portion of their diets comprised of meat relative to the base case. The effect is that blacks are less affected by the change in meat price than in the base case, such that there is less difference

in obesity prevalence and life years lived under various meat price increases compared to the status quo.

Meat Consumption (Fig P and Q)

We explore how our results would change if all individuals' diets were comprised more or less of meats using the upper and lower 95% confidence interval estimates of the proportion of calories from meats for each subpopulation, in accordance with the binomial standard error. Compared to the base case, reduction in the amount of meat calories consumed relative to other calories yielded slightly higher rates of obesity (given that a smaller portion of individuals' calories are impacted by the price change) and less pronounced increase in life years relative to the status quo. Increase in the proportion of calories had the opposite effect.

Gradual Increase in Consumption (Fig R though T)

Given uncertainty about whether the observed trend toward gradual increase in consumption year over year will continue at a rate consistent with historical data (the status quo assumption), we assess how our results change under two alternative scenarios: (1) the increase in daily consumption proceeds at a rate of half that observed historically, and (2) complete cessation of the trend to eat more each year. Reduction in the tendency to gradually increase consumption produced less steep increases in obesity prevalence following the first three years (during which prevalence is strongly influenced by the price increase). This resulted in greater reductions in obesity prevalence over 15 years (Fig R)

than in the base case in which we assumed continuation of the tendency to gradually increase consumption over time (Fig 2). However, the relative difference in obesity prevalence 15 years out under various meat price increases compared to no change in meat price (Fig S) did not differ in these what-if analyses relative to the base case (Fig 3). In comparing life years lived relative to the status quo under a given meat price increase (Fig T), the results for reduced/eliminated tendency to gradually increase consumption were not notably different than in the base case for white males, white females, and black females. However, for black males, reduction in the tendency to increase consumption results in lower life expectancy.

Explicit Modeling of Smoker Status (Fig U and V)

In this analysis, instead of arriving at an average risk ratio for mortality by BMI for individuals of each race-gender group by performing a weighted average of those for smokers and nonsmokers, we assign each individual in the model a smoker status, such that their risk of mortality by BMI accounts for this in addition to their race and gender. This does not impact our results.

Degree of Prevalence Disease (Fig W)

Patel's assessment of risk ratio for mortality by BMI for each race-gender differentiates between those who, at the initiation of the American Cancer Society's Cancer Prevention Study II had one or more of the following conditions: cancer, heart disease, stroke, respiratory disease, current sickness, or weight loss of at least 10 lbs. in the

previous year.[9] Those with any of these conditions are identified by Patel as having "prevalent disease." In this what-if analysis, instead of arriving at a risk ratio for mortality by BMI for individuals of each race-gender group by performing a weighted average of those for individuals with and without illness, we perform the simulation under both of these extreme scenarios: (1) no prevalent disease in the population, and (2) 100% of the population with prevalent disease. In scenario A, the effect of meat price increase on life years lived was not notably different from the base case. In scenario B, increasing meat price resulted in smaller increases in life expectancy than in the base case and decreased life expectancy for black males. Discrepancies by race-gender group remain.

Effect of Substitution/Complements (Fig X and Y)

Cross price elasticity data suggests that an increase in meat price would result in reduction in the consumptions of many other types of food in addition to meats. If we account for changes in consumption of non-meats in addition to meats, the effect of meat price increase on obesity prevalence and life years lived is more dramatic than in the base case, with further reductions in obesity prevalence relative to no meat price change (Fig S24). This results in additional life years lived relative to the base case for white males, white females, and black females. Black males, however receive less mortality benefit than in the base case, and are harmed if meat prices increase by 50%.

Price Elasticity of Demand for Meats (Fig Z and AA)

We assess the impact of meat price elasticity of demand values over a wide range by executing our model with elasticity of demand for red meats, white meats, and seafood at (1) half and (2) double their base case values. Halving the elasticities resulted in less dramatic reductions in obesity prevalence relative to the status quo, while doubling it yielded drastic reductions in prevalence (Fig Z). Halved elasticity of demand yielded smaller increases in life years relative to the base case (Fig AA). The extreme reductions in obesity prevalence assuming doubled elasticity would be less beneficial for black males than the base case (and posed harm relative to the status quo for the largest price increases examined), but benefited the other subpopulations.

Price Increase Confined to Specific Meat Types (Fig BB through EE)

Our base analysis assumes price increase across all types of meat. While it is likely that, due to substitution effects, the price of all meats would rise in response to a sustained increase in the price of one type of meat (e.g., red meats), here we evaluate the effect of a price increase confined to red meat alone (Fig BB and CC) and seafood alone (Fig DD and EE). In both of these cases the results are similar to the base case, but scaled to reflect the smaller portion of diet made up by red meat alone or seafood alone.

Price Decrease (Fig FF and GG)

We also assessed the impact of a 5%, 10%, 25%, or 50% *reduction* in meat price. Larger decreases in price resulted in more dramatic increases in obesity prevalence and reductions in life years. For the largest price reductions, the magnitudes of the decrease in

life years were greater than that for corresponding increases in life years gained under price increases. While in the case of price increases, the tendency to gradually increase consumption worked in the opposite direction of the price change, in the case of price decreases, both effects are in the same direction.

Appendix Figures

Fig A. Initial BMI Distribution. Initial distribution of BMIs for each race-gender.

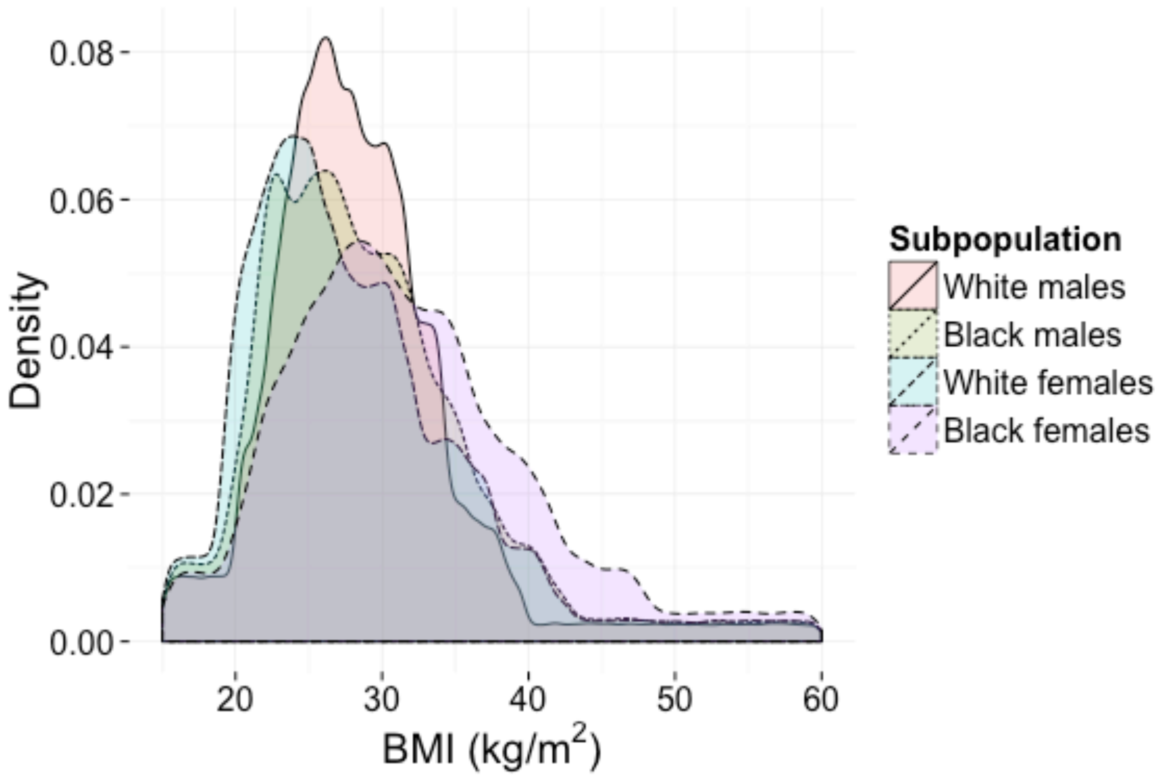

Fig B. Risk of Death by BMI. Relative risk of death for specified BMI ranges relative to 22.5-25.0 kg/m^2 by race-gender.

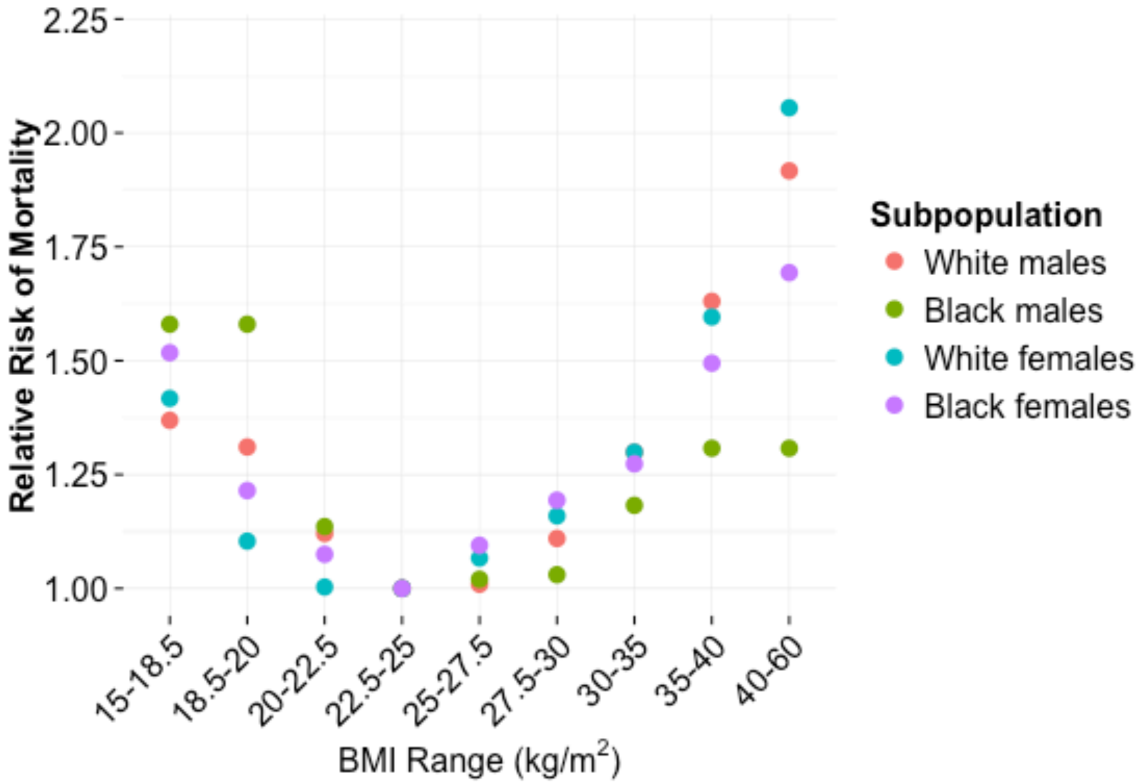

Fig C. Obesity Prevalence Over Time. Prevalence of obesity (BMI ≥30 kg/m²) over 15 years by race-gender, for each meat price increase.

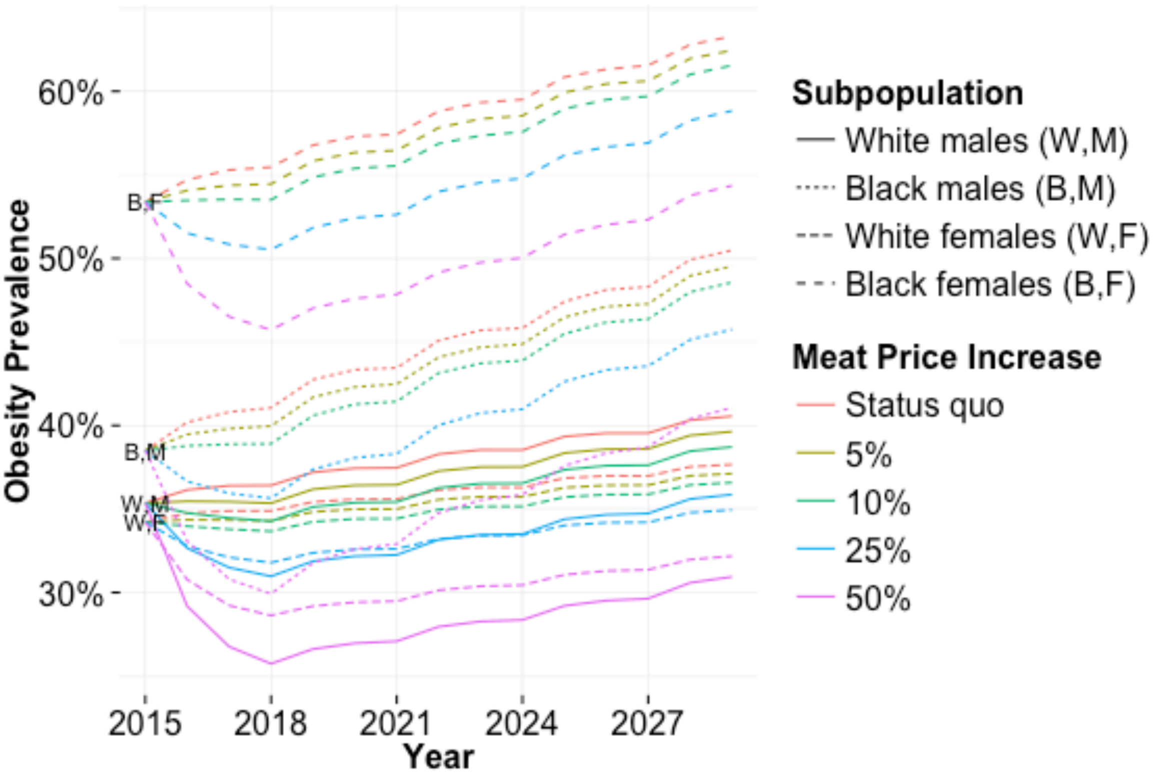

Fig D. Overweight Prevalence Over Time. Prevalence of overweight (BMI ≥25 kg/m^2) over 15 years by race-gender, for each meat price increase.

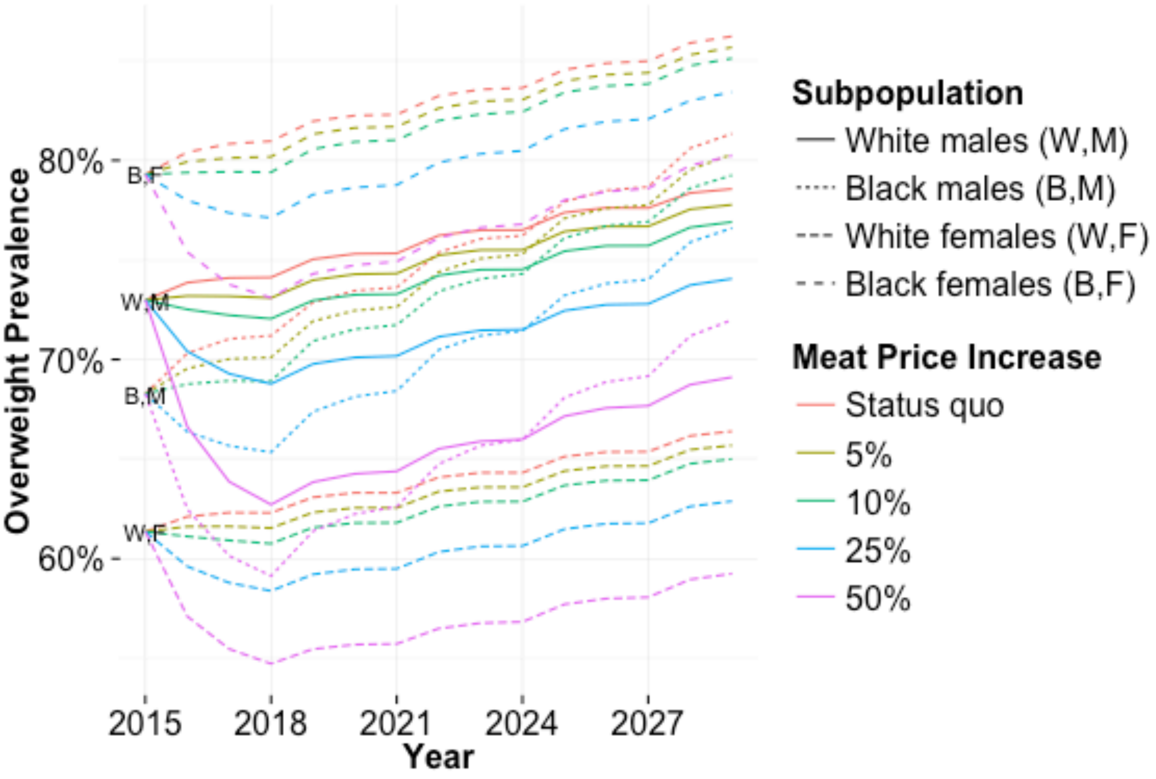

Fig E. Underweight Prevalence Over Time. Prevalence of underweight (BMI <18.5 kg/m^2) over 15 years by race-gender, for each meat price increase.

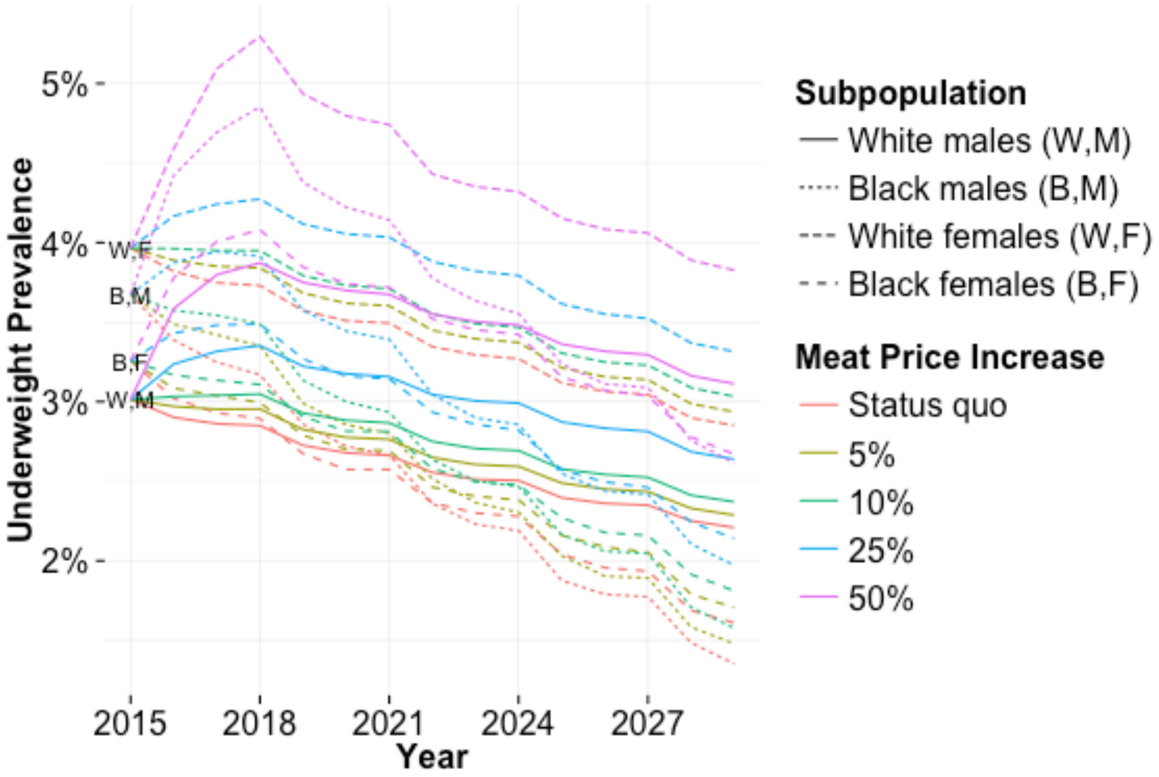

Fig F. Difference in Quality Adjusted Life Years Lived Over 15 Years Relative to Status Quo. (1) Difference in quality adjusted life years lived over 15 years per 1000 people under specified meat price increase, relative to that under no price change. (2) Same as 1, including only individuals with initial BMI greater than or equal to 25 kg/m2. (3) Same as 1, including only individuals with initial BMI less than 25 kg/m^2.

1) 2) 3)

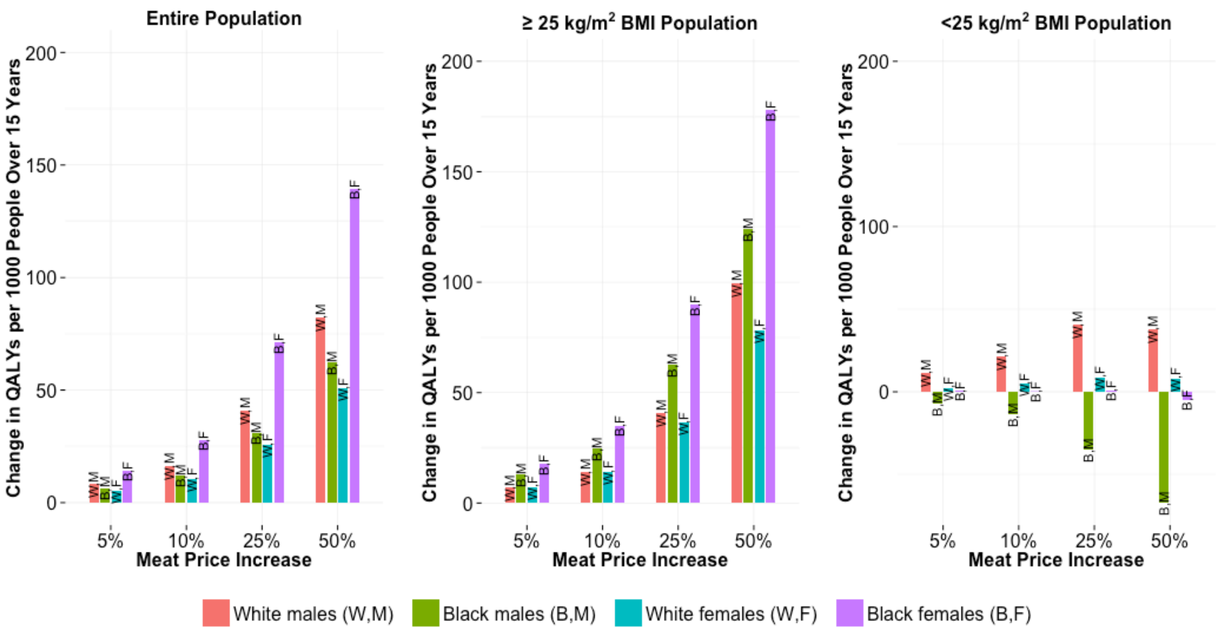

Fig G. Difference in Life Years Relative to Status Quo, Equal RRs of Mortality Across Races. Difference in life years lived over 15 years per 1000 people under specified meat price increase, relative to that under no price change, given black risk ratios of mortality by BMI equal to that for whites.

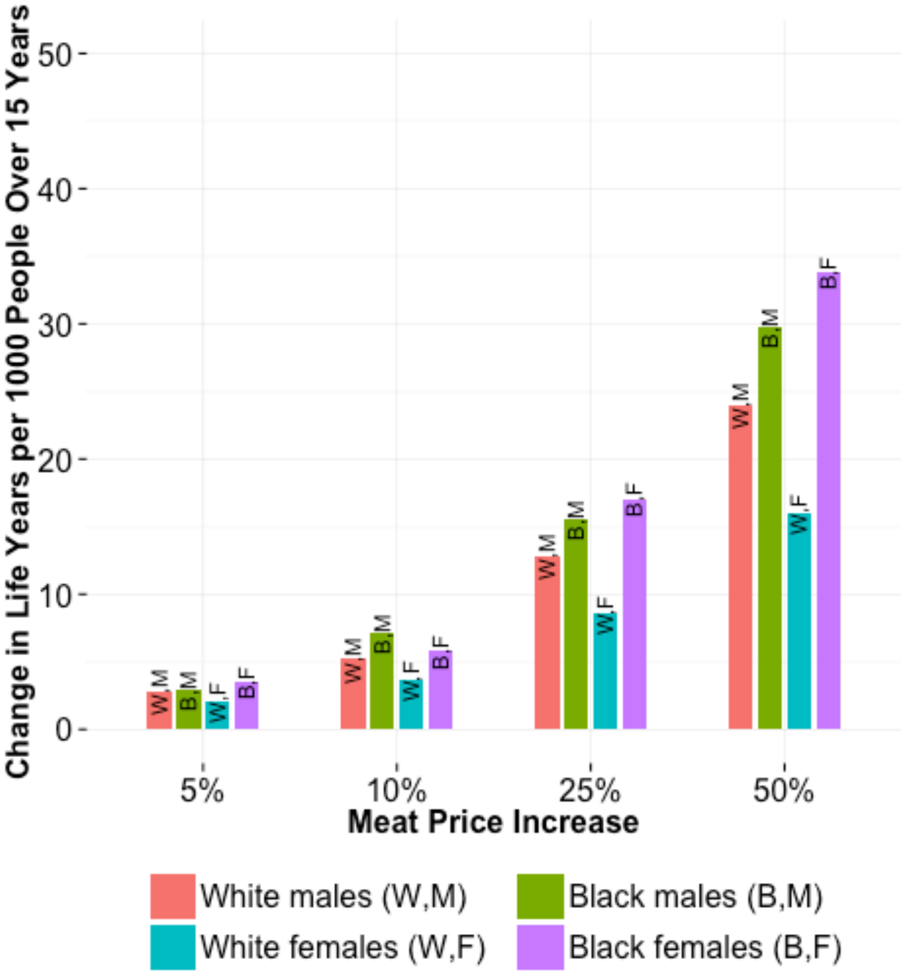

Fig H. Difference in Life Years Relative to Status Quo, Reduced Black Male RR of Mortality for 15-18.5 and 18.5-20 kg/m^2. Difference in life years lived over 15 years per 1000 people under specified meat price increase, relative to that under no price change, given black male risk ratios of mortality for 15-18.5 and 18.5-20 kg/m^2 reduced to that for 22.5-25 kg/m^2.

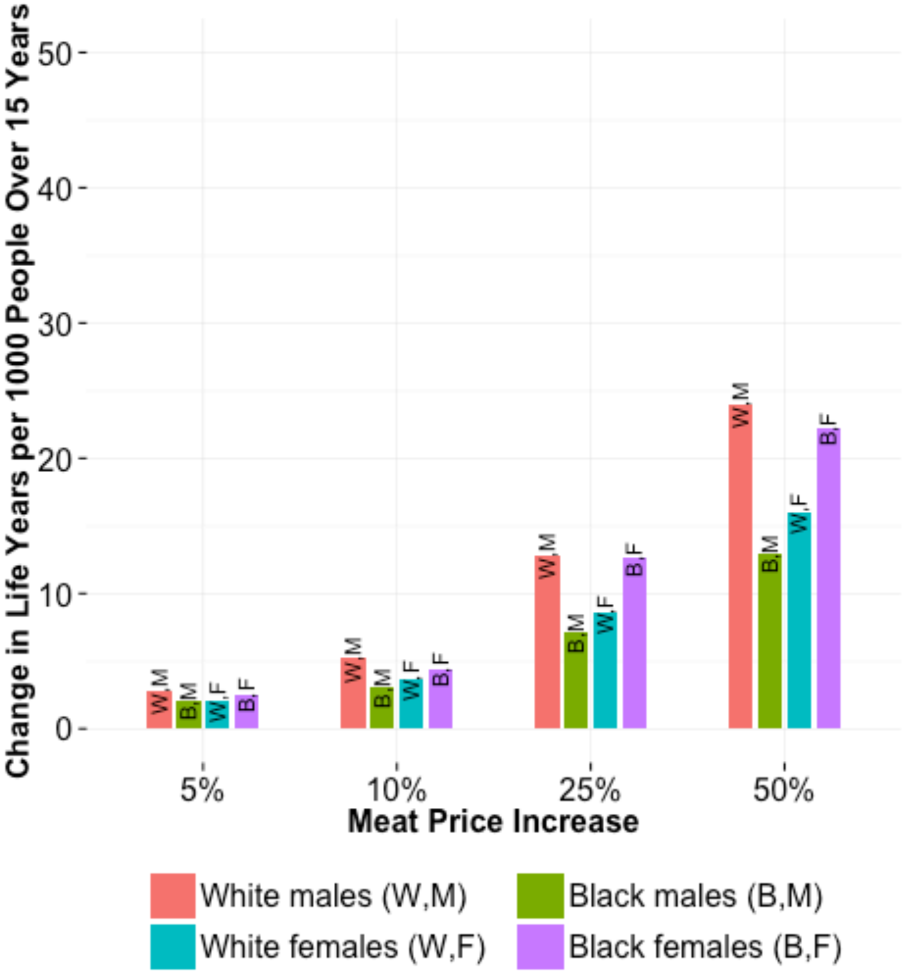

Fig I. Difference in Life Years Relative to Status Quo, Reduced Black Male RR of Mortality for 18.5-20 kg/m². Difference in life years lived over 15 years per 1000 people under specified meat price increase, relative to that under no price change, given black male risk ratios of mortality for 18.5-20 kg/m² reduced to that for 22.5-25 kg/m².

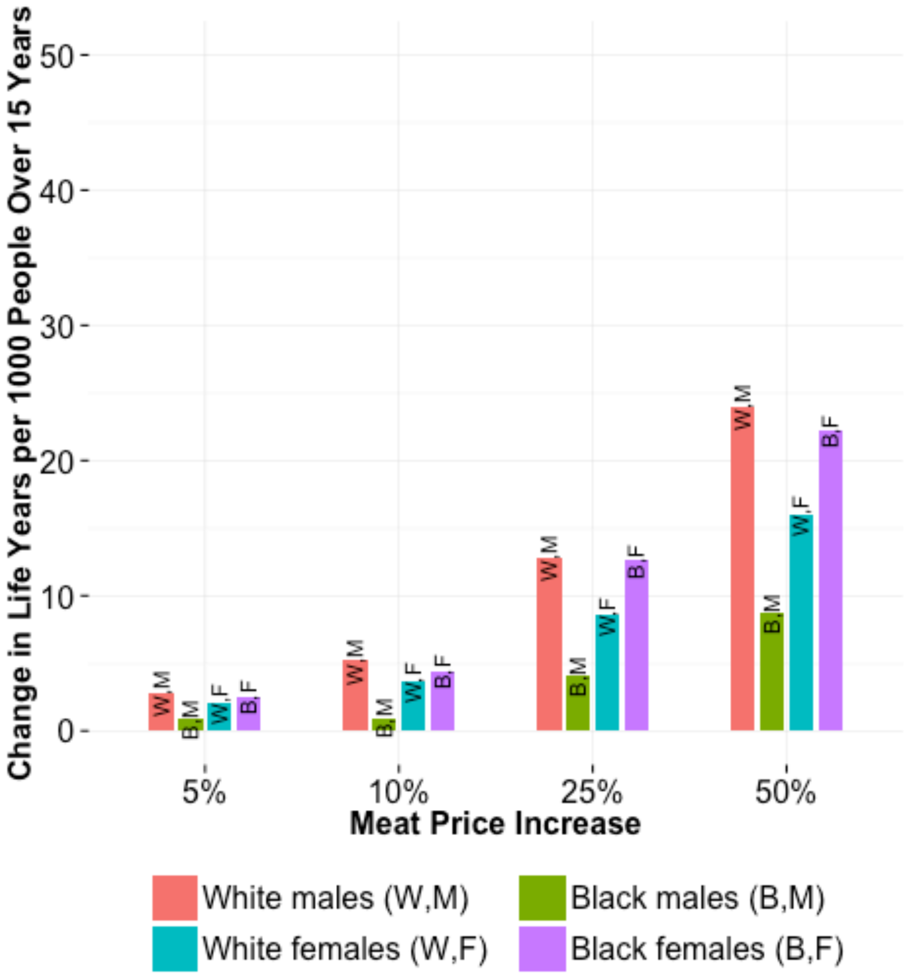

Fig J. Difference in Life Years Relative to Status Quo, Increased Black Male RR of Mortality for ≥40 kg/m². Difference in life years lived over 15 years per 1000 people under specified meat price increase, relative to that under no price change, given black male risk ratios of mortality for BMI ≥40 kg/m² increased to that for white males.

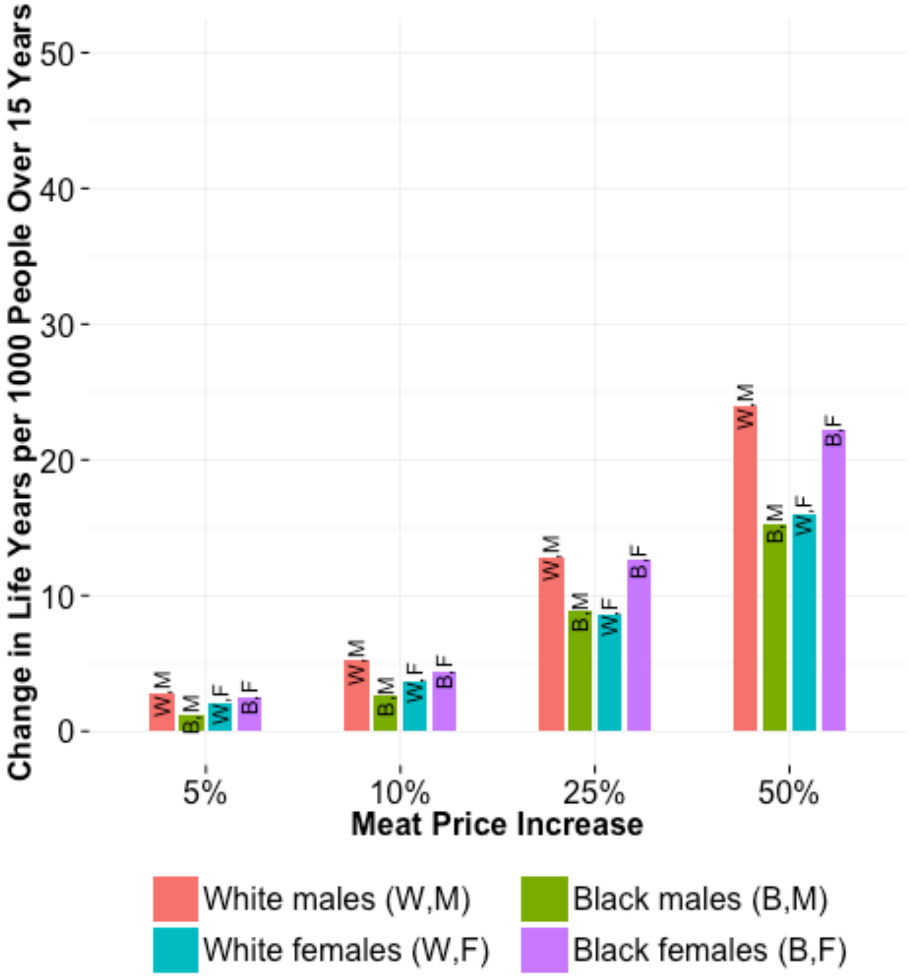

Fig K. Difference in Life Years Relative to Status Quo, No Increased Risk of Mortality Below 25kg/m^2 BMI. Difference in life years lived over 15 years per 1000 people under specified meat price increase, relative to that under no price change, given no increase in relative risk of mortality for BMIs below 25 kg/m^2.

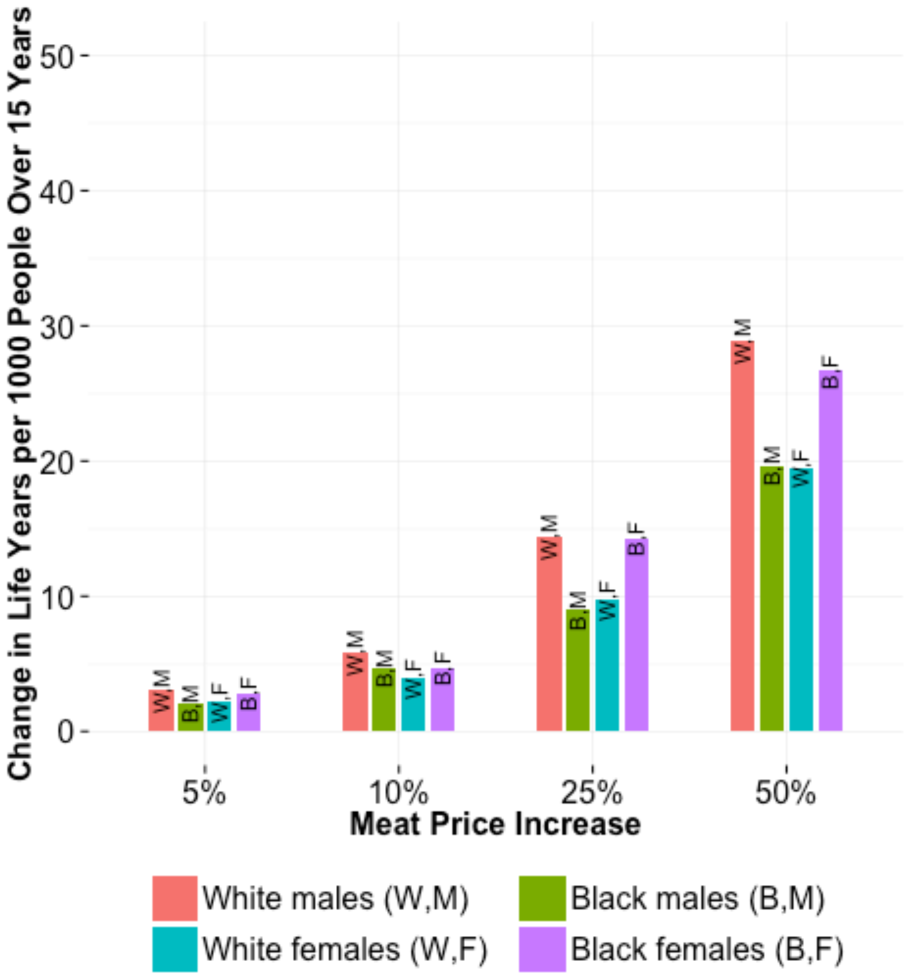

Fig L. Difference in Obesity Prevalence after 15 Years Relative to Status Quo, Black Initial BMI Distributions Equal to White. Difference in 2030 obesity prevalence under specified meat price increase, relative to 2030 prevalence under no price change, given black initial BMI distributions equal to that of whites of respective gender.

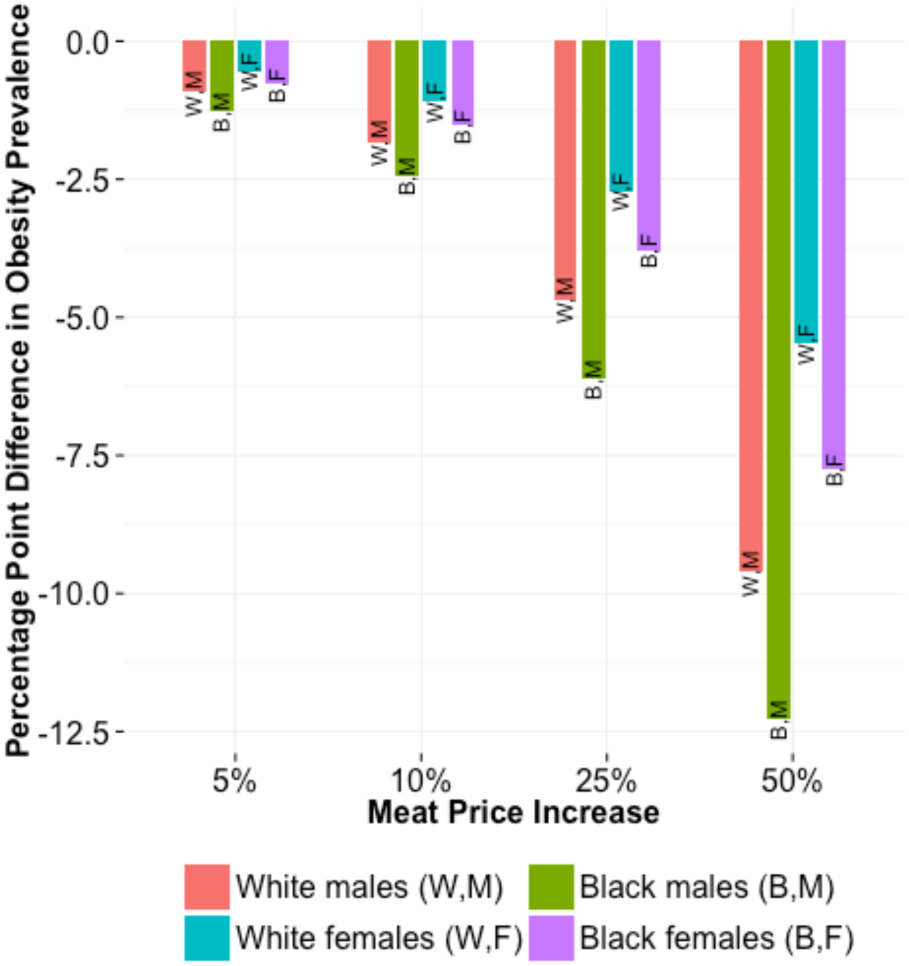

Fig M. Difference in Life Years Relative to Status Quo, Black Initial BMI Distributions Equal to White.

Difference in life years lived over 15 years per 1000 people under specified meat price increase, relative to that under no price change, given black initial BMI distributions equal to that of whites of respective gender.

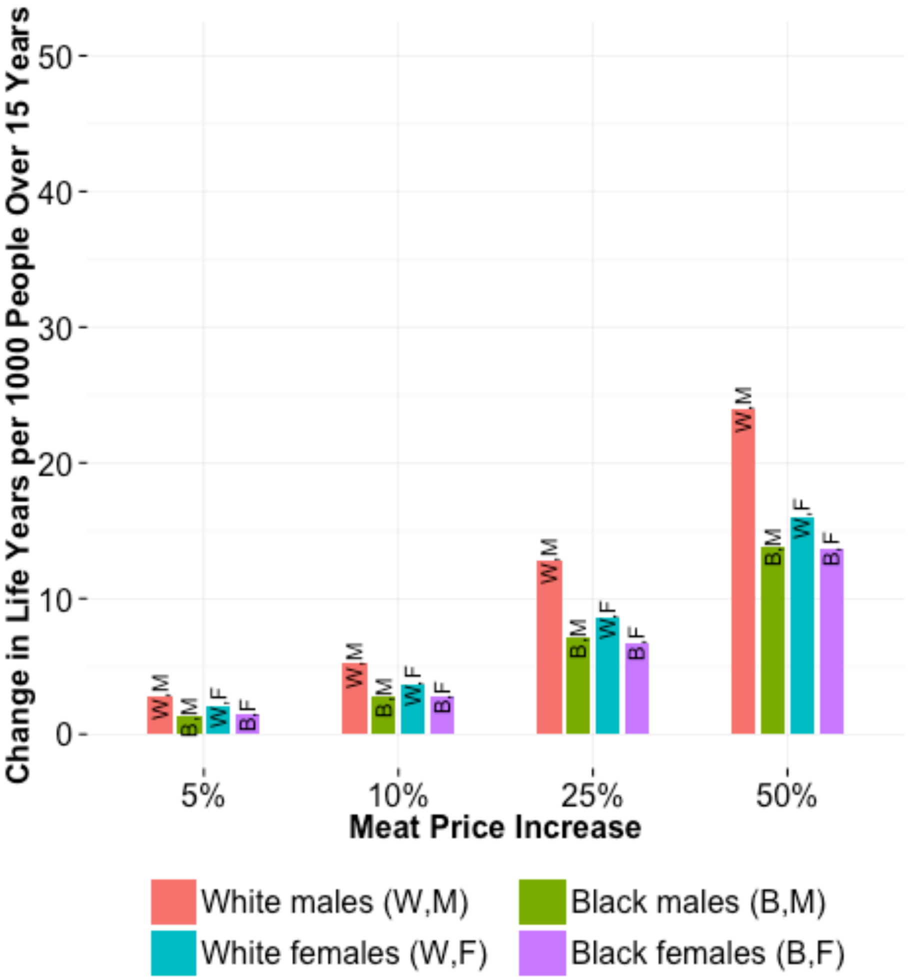

Fig N. Difference in Obesity Prevalence after 15 Years Relative to Status Quo, Black Food Consumption Pattern Same as White. Difference in 2030 obesity prevalence under specified meat price increase, relative to 2030 prevalence under no price change, given proportion of calories from meats for blacks equal to that for whites by respective gender.

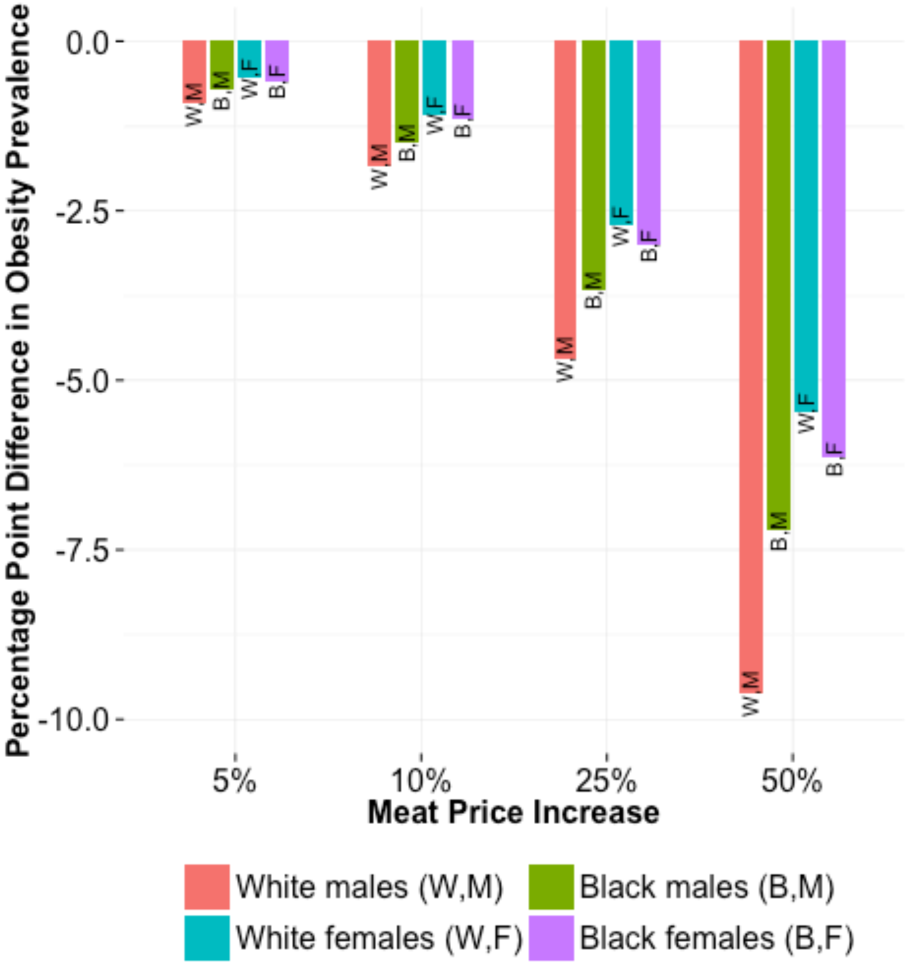

Fig O. Difference in Life Years Relative to Status Quo, Black Food Consumption Pattern Same as White. Difference in life years lived over 15 years per 1000 people under specified meat price increase, relative to that under no price change, given proportion of calories from meats for blacks equal to that for whites by respective gender.

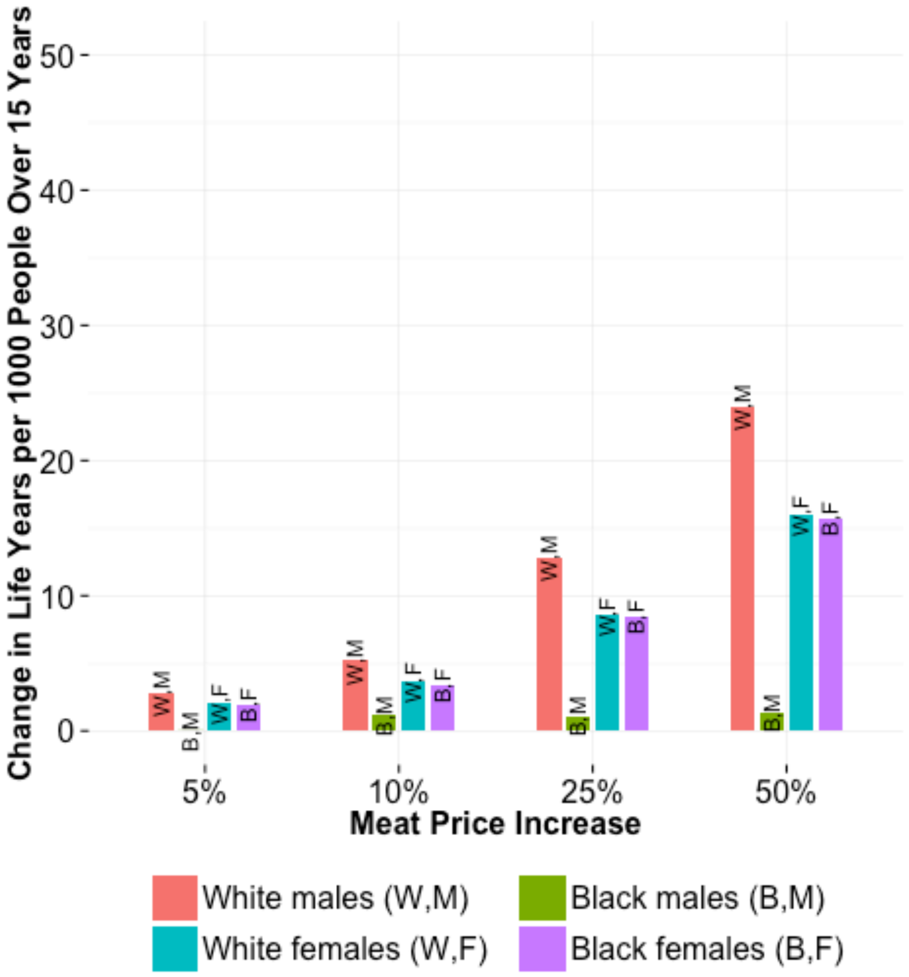

Fig P. Difference in Obesity Prevalence after 15 Years Relative to Status Quo, Lower or Upper 95% CI Meat Consumption (1) Difference in 2030 obesity prevalence under specified meat price increase, relative to 2030 prevalence under no price change, given proportion of calories at lower 95th percentile. (2) Difference in 2030 obesity prevalence under specified meat price increase, relative to 2030 prevalence under no price change, given proportion of calories at upper 95th percentile.

1)

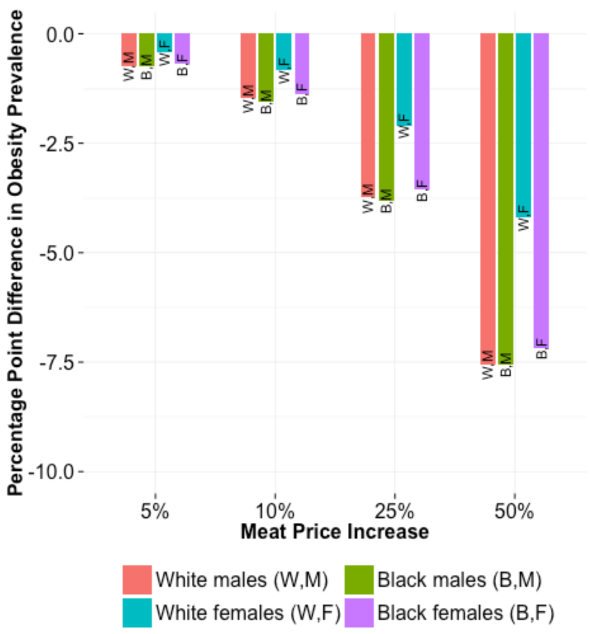

2)

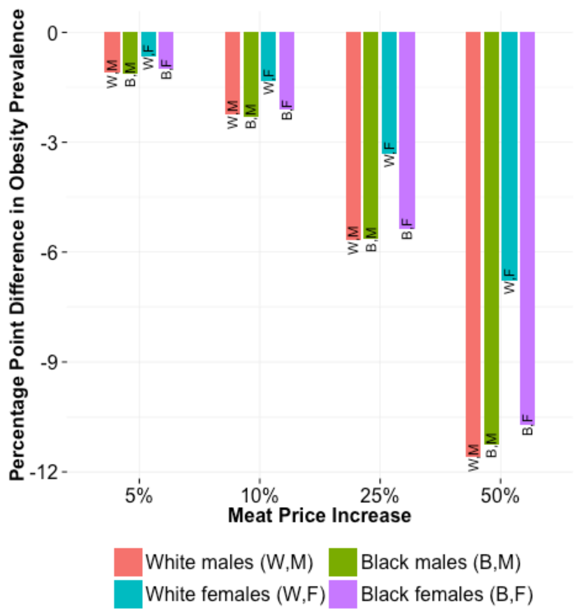

Fig Q. Difference in Life Years Relative to Status Quo, Lower or Upper 95% CI Meat Consumption (1) Difference in life years lived over 15 years per 1000 people under specified meat price increase, relative to that under no price change, given proportion of calories at lower 95th percentile. (2) Difference in life years lived over 15 years per 1000 people under specified meat price increase, relative to that under no price change, given proportion of calories at upper 95th percentile.

1) 2)

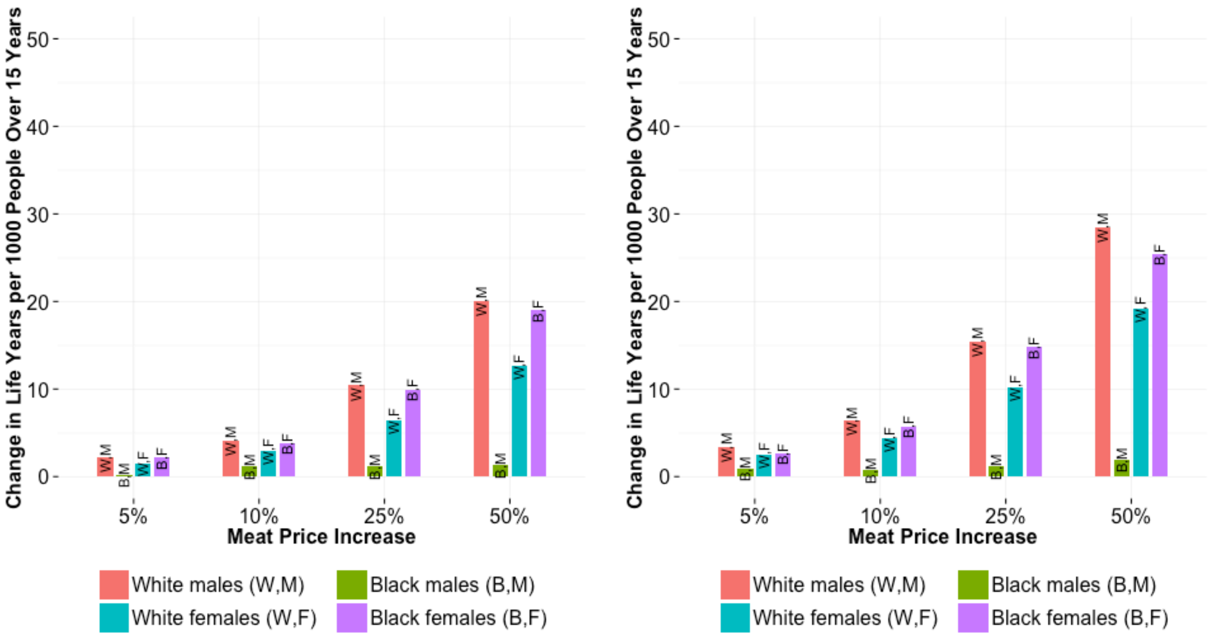

Fig R. Change in Obesity Prevalence Over 15 Years Relative to Current Prevalence, Halved or No Gradual Increase in Consumption. (1) Change in obesity prevalence by race-gender from 2015 to 2030 under each meat price increase, given year-over-year increase in daily caloric consumption reduced by half. (2) Change in obesity prevalence by race-gender from 2015 to 2030 under each meat price increase, given no year-over-year increase in daily caloric consumption.

1)

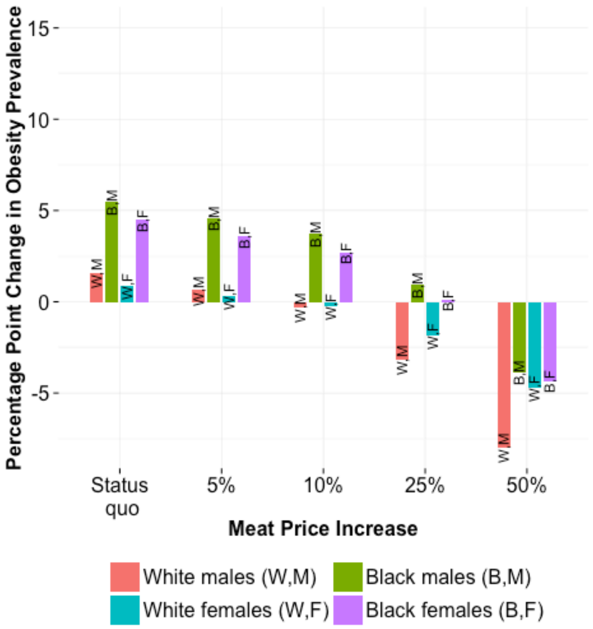

2)

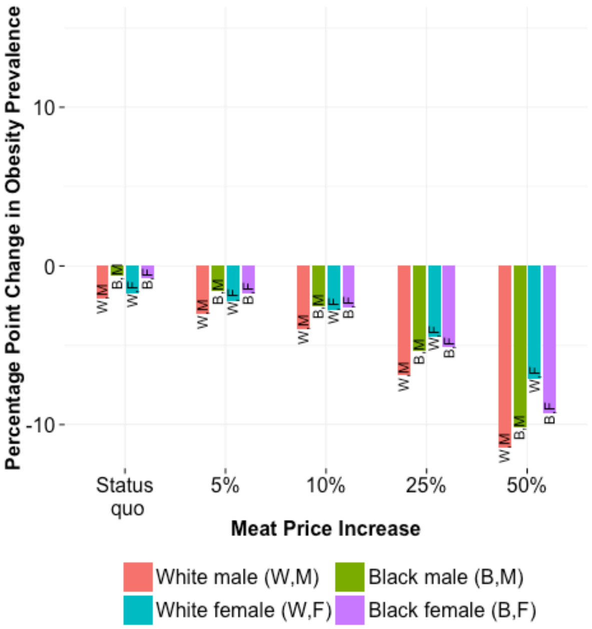

Fig S. Difference in Obesity Prevalence after 15 Years Relative to Status Quo, Halved or No Gradual Increase in Consumption. (1) Difference in 2030 obesity prevalence under specified meat price increase, relative to 2030 prevalence under no price change, given year-over-year increase in daily caloric consumption reduced by half. (2) Difference in 2030 obesity prevalence under specified meat price increase, relative to 2030 prevalence under no price change, given no year-over-year increase in daily caloric consumption.

1)

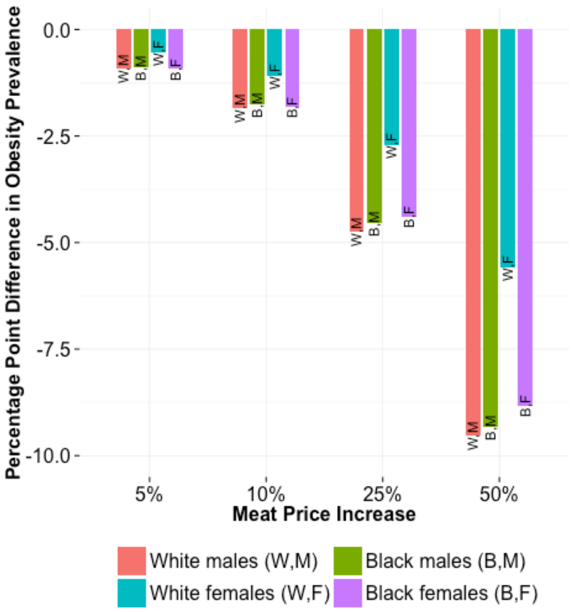

2)

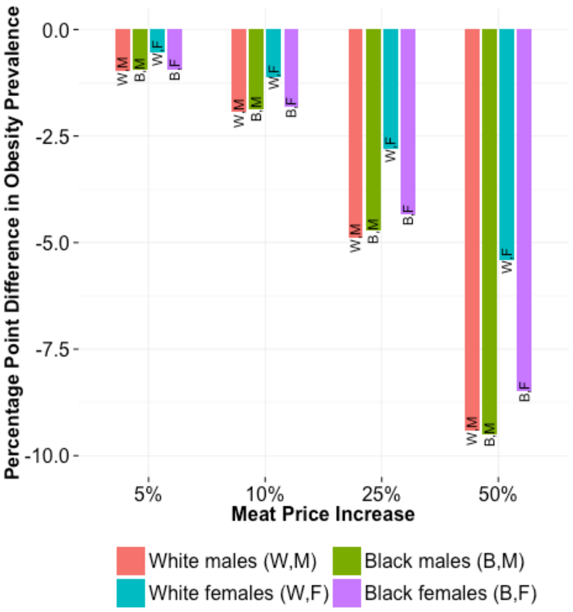

Fig T. Difference in Life Years Relative to Status Quo, Halved or No Gradual Increase in Consumption.

(1) Difference in life years lived over 15 years per 1000 people under specified meat price increase, relative to that under no price change, given year-over-year increase in daily caloric consumption reduced by half. (2) Difference in life years lived over 15 years per 1000 people under specified meat price increase, relative to that under no price change, given no year-over-year increase in daily caloric consumption.

1)

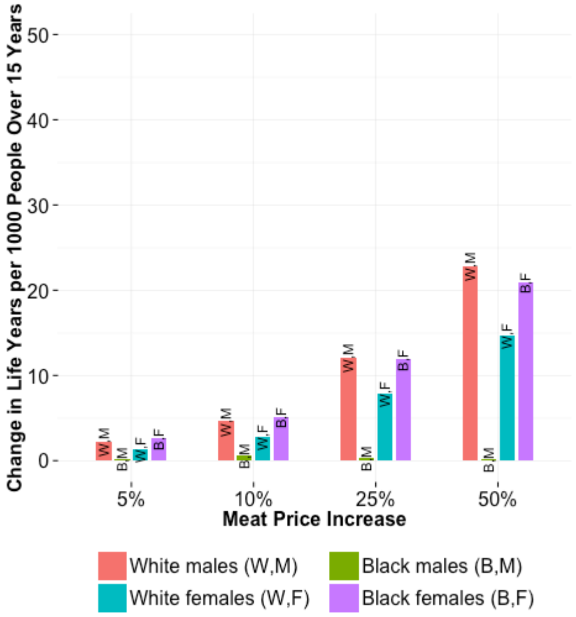

2)

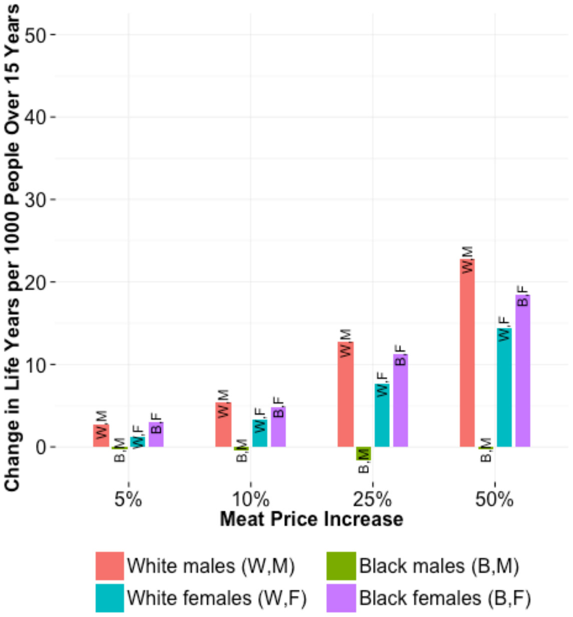

Fig U. Difference in Obesity Prevalence after 15 Years Relative to Status Quo, Explicit Modeling of Smoker Status. Difference in 2030 obesity prevalence under specified meat price increase, relative to 2030 prevalence under no price change, given individuals' smoker status modeled explicitly.

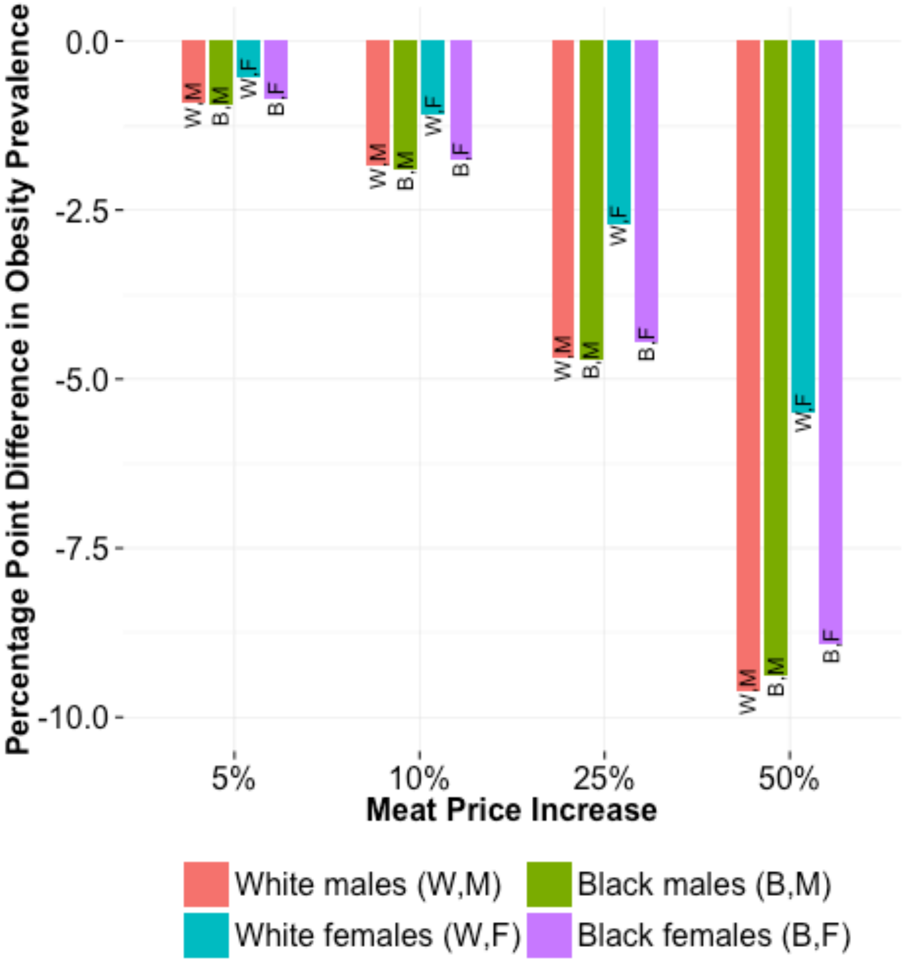

Fig V. Difference in Life Years Relative to Status Quo, Explicit Modeling of Smoker Status. Difference in life years lived over 15 years per 1000 people under specified meat price increase, relative to that under no price change, given individuals' smoker status modeled explicitly.

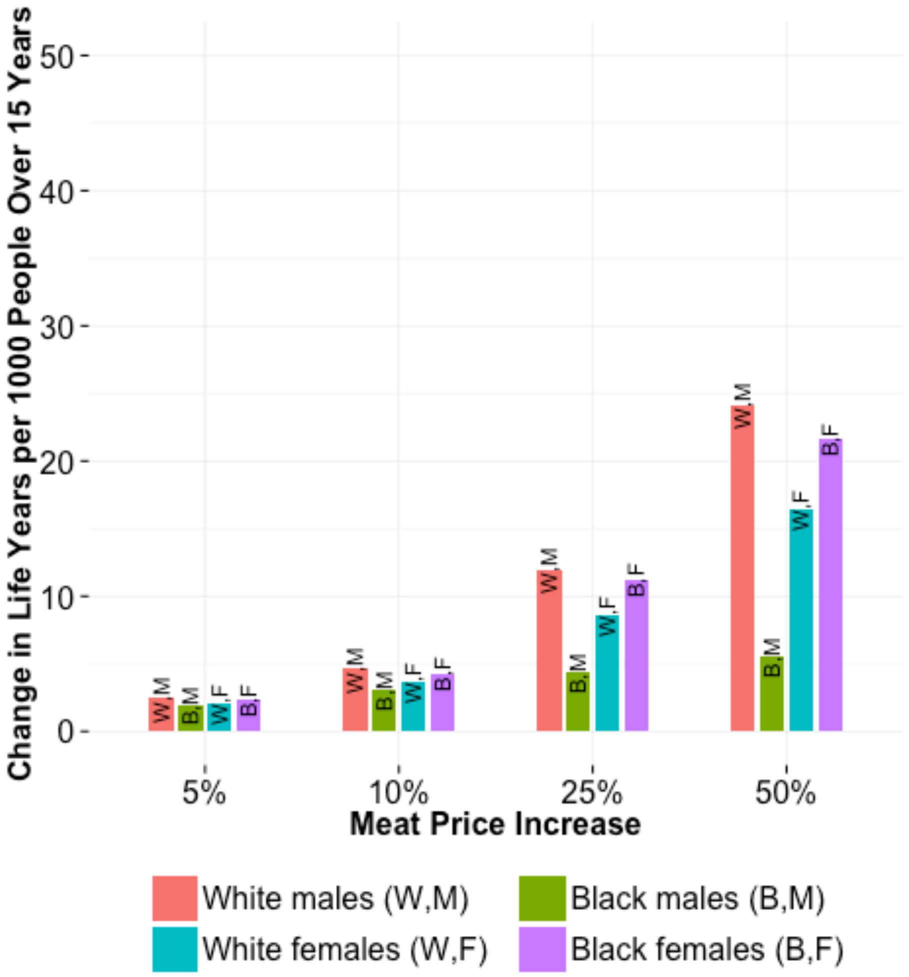

Fig W. Difference in Life Years Relative to Status Quo, Varying Prevalence of Illness (1) Difference in life years lived over 15 years per 1000 people under specified meat price increase, relative to that under no price change, given 0% illness prevalence. (2) Difference in life years lived over 15 years per 1000 people under specified meat price increase, relative to that under no price change, given 100% illness prevalence.

1)

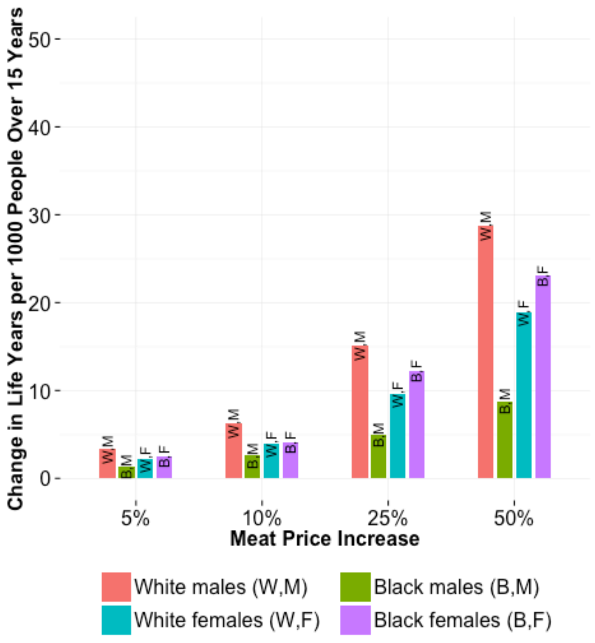

2)

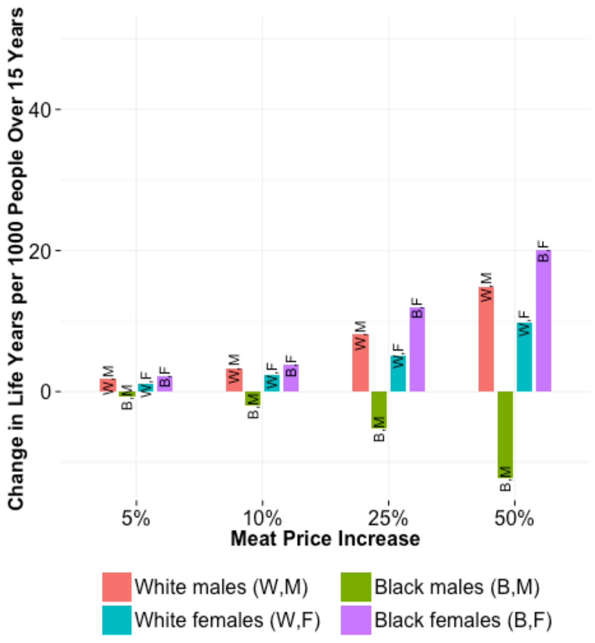

Fig X. Difference in Obesity Prevalence after 15 Years Relative to Status Quo, Accounting for Complement/Substitution Effects. Difference in 2030 obesity prevalence under specified meat price increase, relative to 2030 prevalence under no price change, given accounting for effect of complements/substitutes for meats.

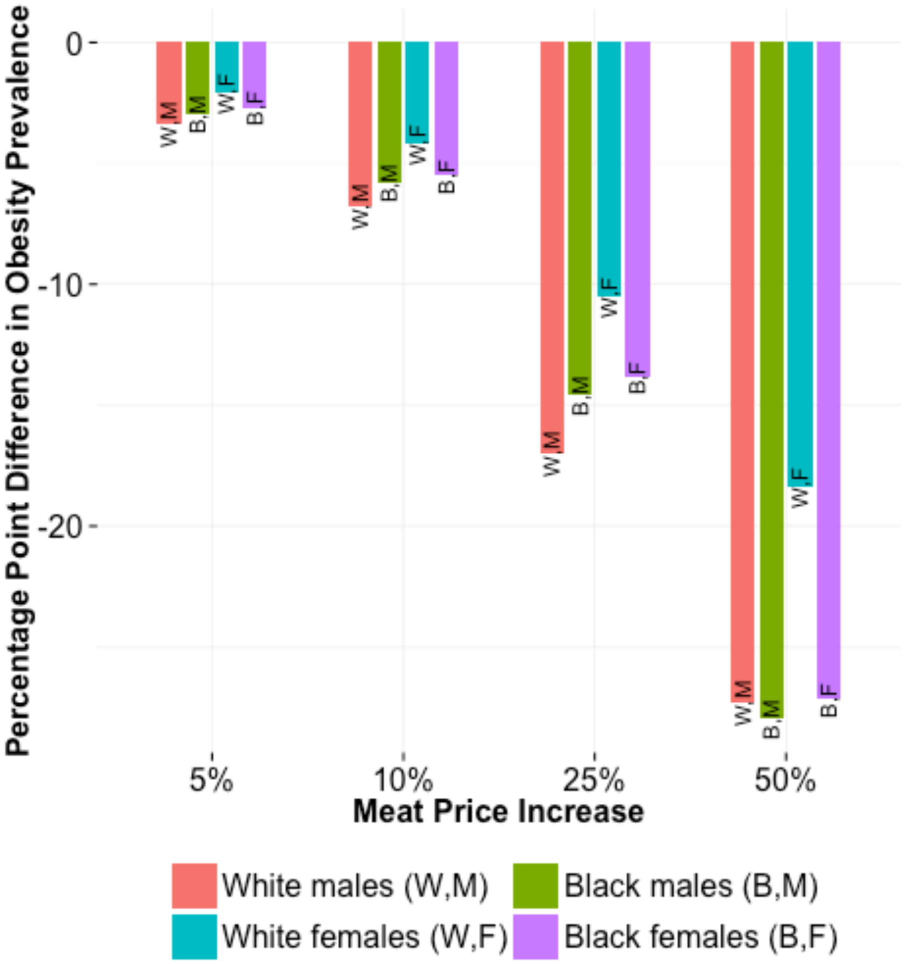

Fig Y. Difference in Life Years Relative to Status Quo, Accounting for Complement/Substitution Effects. Difference in life years lived over 15 years per 1000 people under specified meat price increase, relative to that under no price change, given accounting for effect of complements/substitutes for meats.

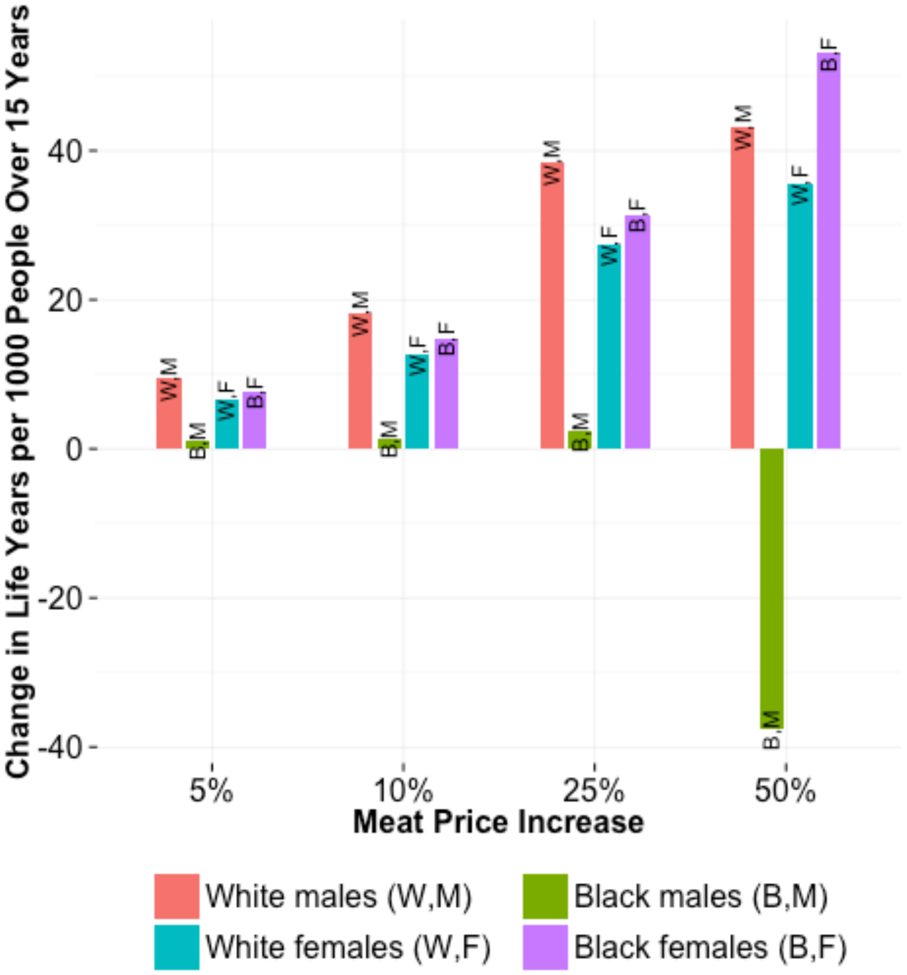

Fig Z. Difference in Obesity Prevalence after 15 Years Relative to Status Quo, Varying Meat Price Elasticity of Demand. (1) Difference in 2030 obesity prevalence under specified meat price increase, relative to 2030 prevalence under no price change, given halved price elasticity of demand for meats. (2) Difference in 2030 obesity prevalence under specified meat price increase, relative to 2030 prevalence under no price change, given doubled price elasticity of demand for meats.

1)

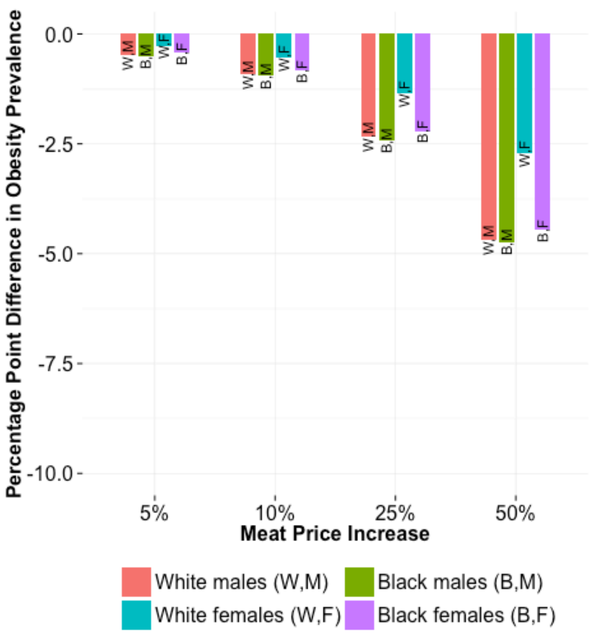

2)

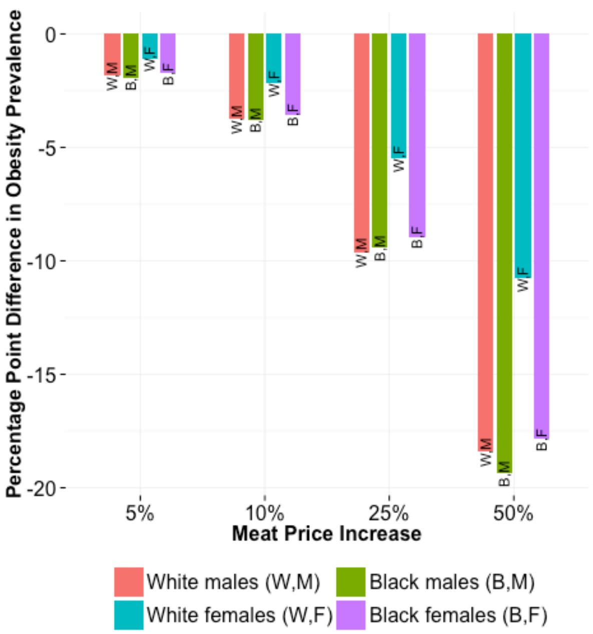

Fig AA. Difference in Life Years Relative to Status Quo, Varying Meat Price Elasticity of Demand. (1) Difference in life years lived over 15 years per 1000 people under specified meat price increase, relative to that under no price change, given halved price elasticity of demand for meats. (2) Difference in life years lived over 15 years per 1000 people under specified meat price increase, relative to that under no price change, given doubled price elasticity of demand for meats.

1)

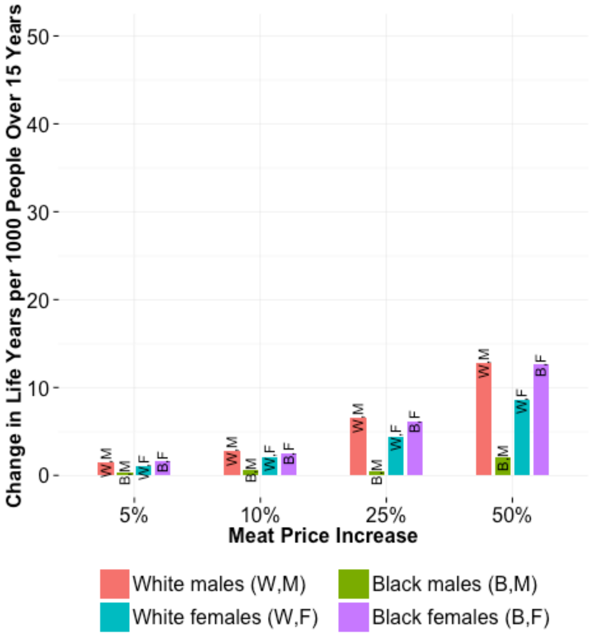

2)

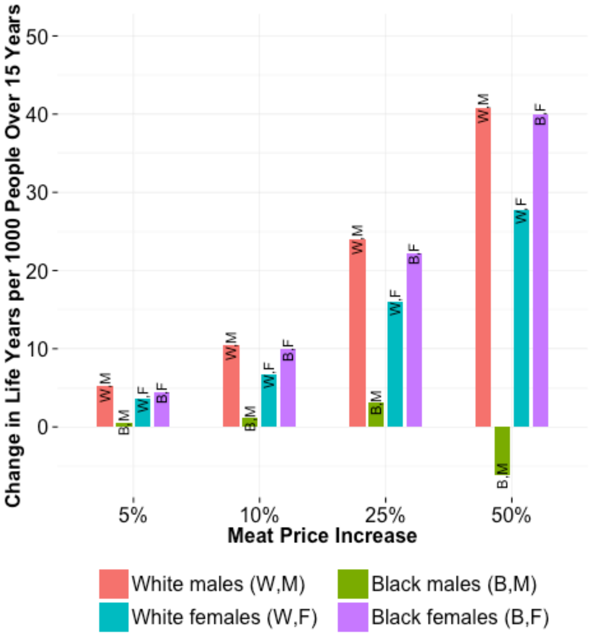

Fig BB. Difference in Obesity Prevalence after 15 Years Relative to Status Quo, Increase in Red Meat Price Only. Difference in 2030 obesity prevalence under specified red meat price increase, relative to 2030 prevalence under no price change.

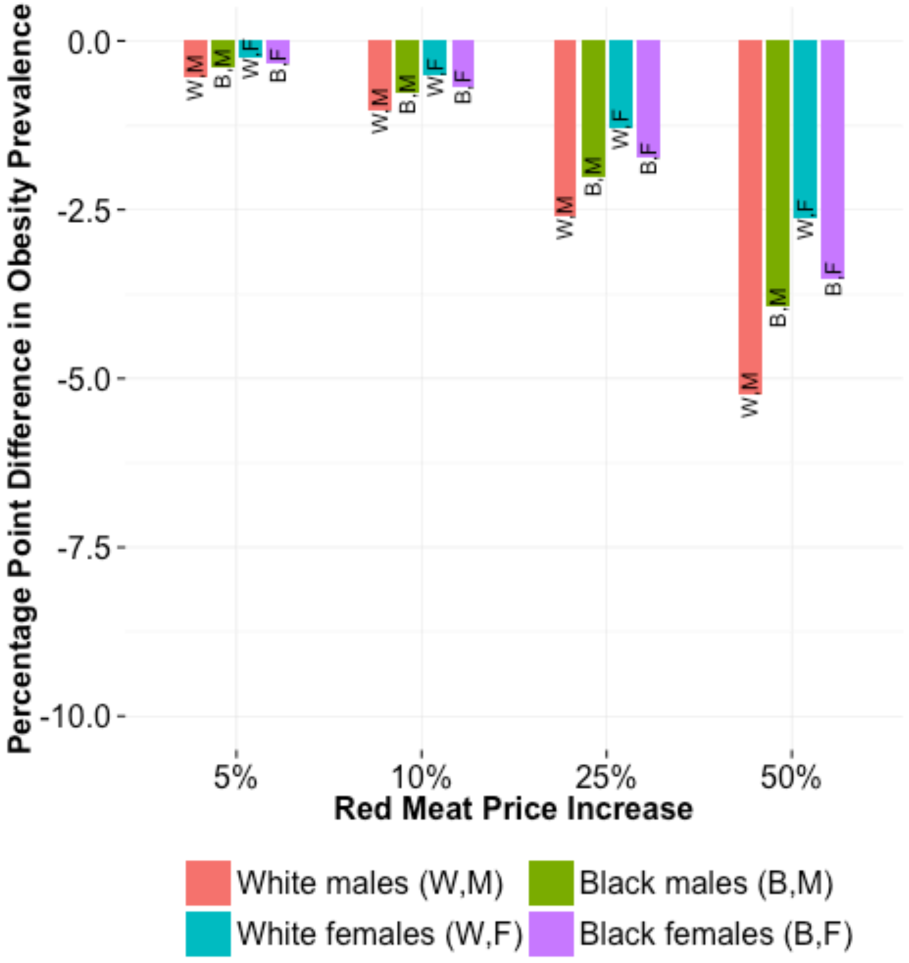

Fig CC. Difference in Life Years Relative to Status Quo, Increase in Red Meat Price Only. Difference in life years lived over 15 years per 1000 people under specified red meat price increase, relative to that under no price change.

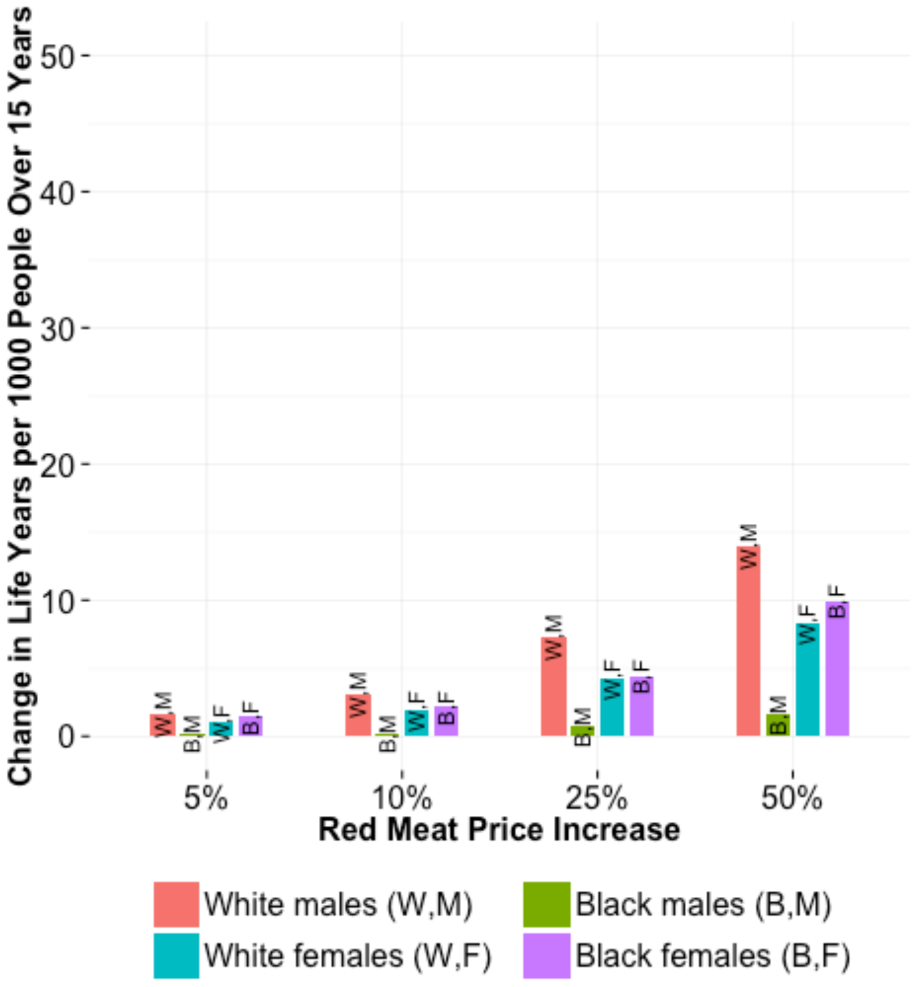

Fig DD. Difference in Obesity Prevalence after 15 Years Relative to Status Quo, Increase in Seafood Price Only. Difference in 2030 obesity prevalence under specified seafood price increase, relative to 2030 prevalence under no price change.

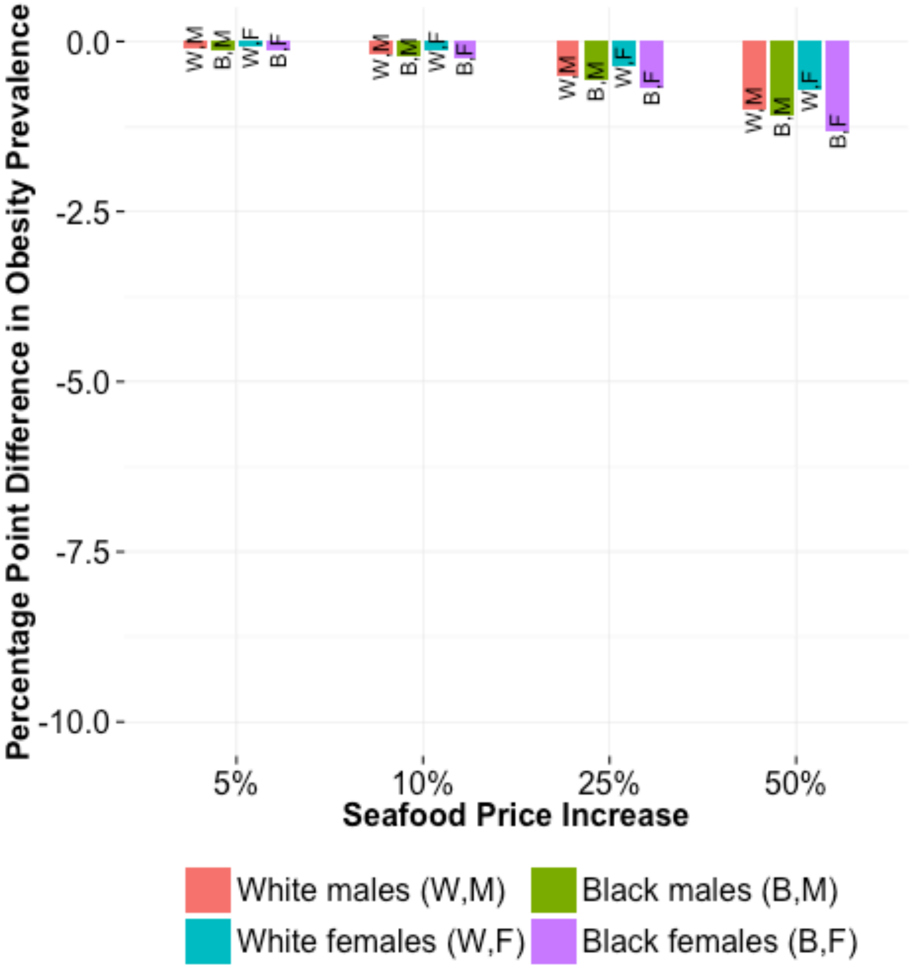

Fig EE. Difference in Life Years Relative to Status Quo, Increase in Seafood Price Only. Difference in life years lived over 15 years per 1000 people under specified seafood price increase, relative to that under no price change.

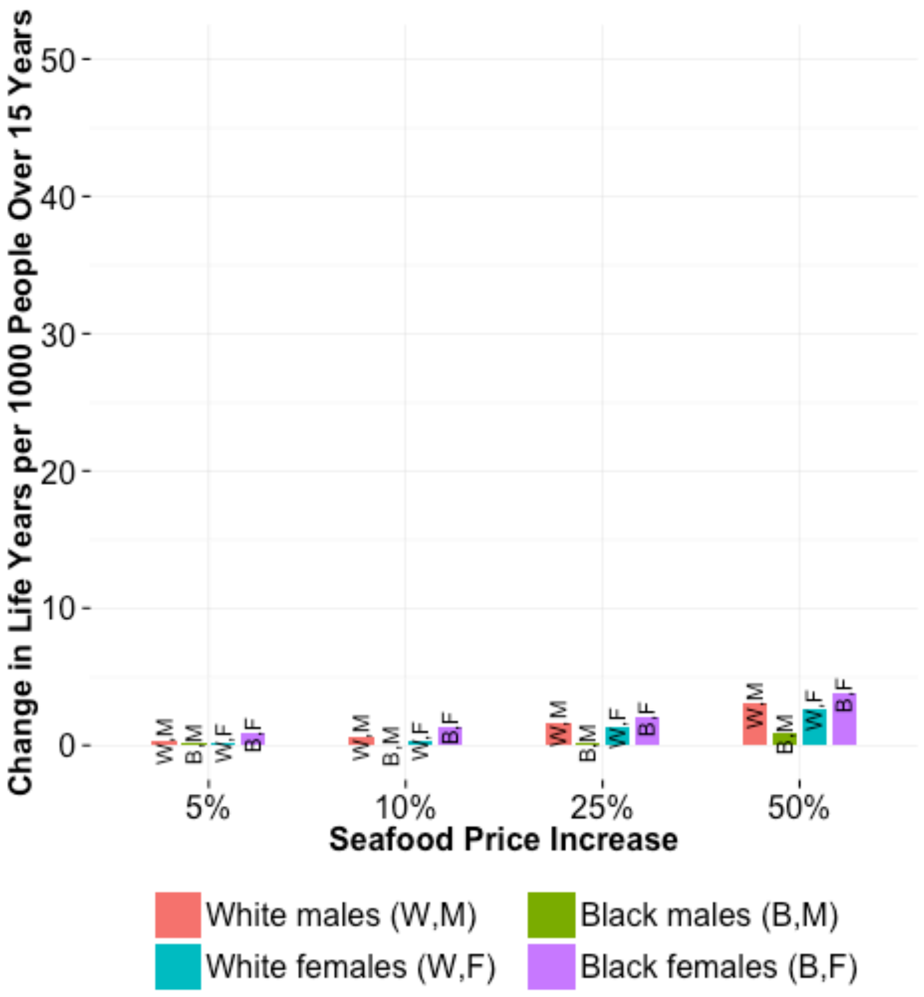

Fig FF. Difference in Obesity Prevalence after 15 Years Relative to Status Quo, Meat Price Decreases.

Difference in 2030 obesity prevalence under specified meat price decrease, relative to 2030 prevalence under no price change.

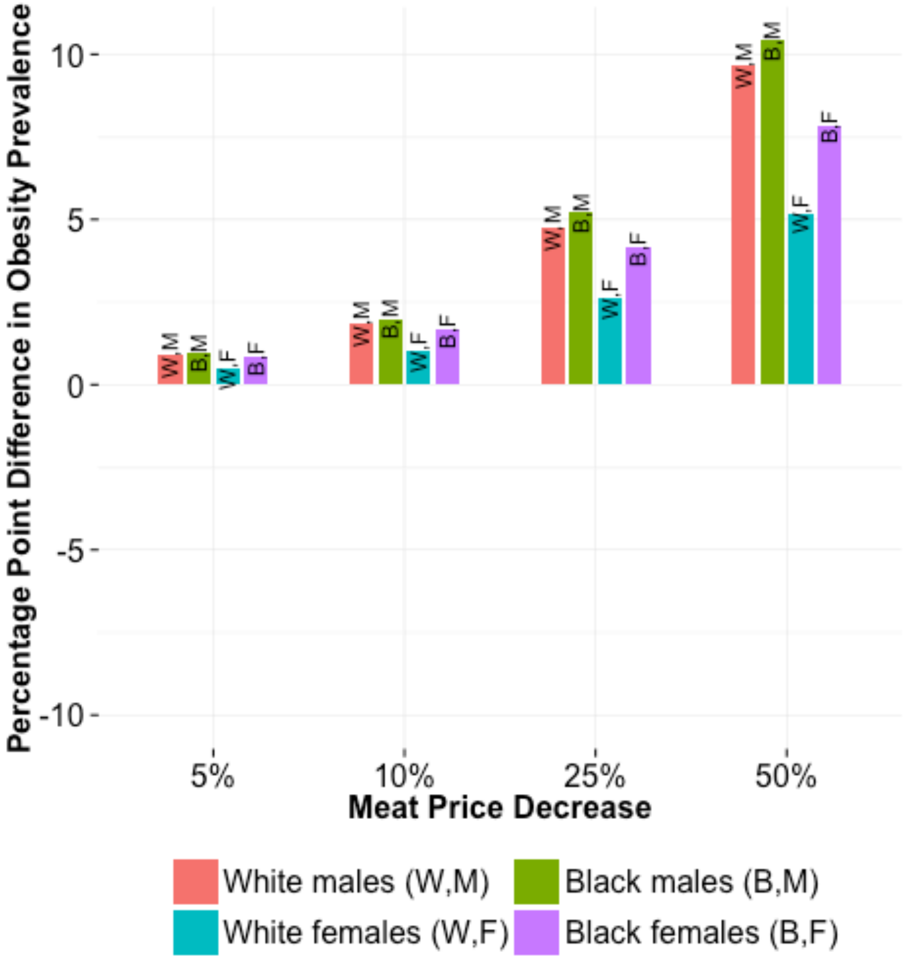

Fig GG. Difference in Life Years Relative to Status Quo, Meat Price Decreases. Difference in life years lived over 15 years per 1000 people under specified meat price decrease, relative to that under no price change.

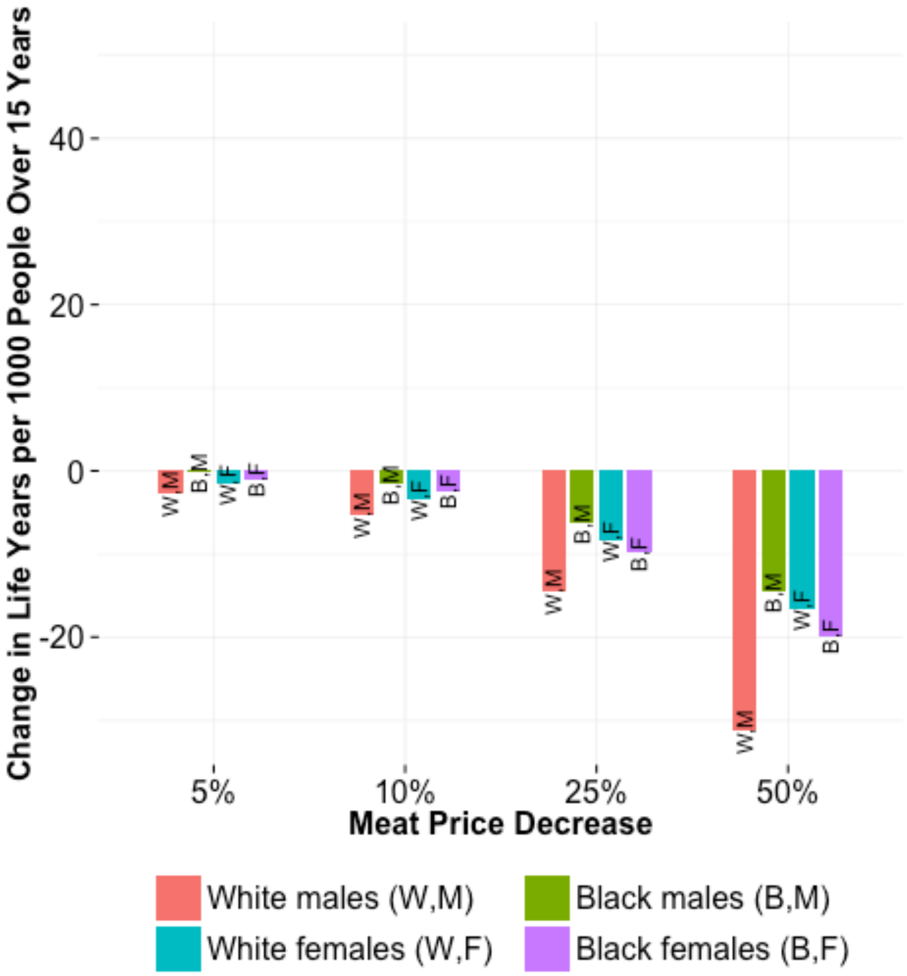

References

1. Fryar CD, Gu Q, Ogden CL. Anthropometric Reference Data for Children and Adults: United States, 2007–2010. Vital Health Stat. 2012;11(252).

2. The Henry J Kaiser Family Foundation. Number of Deaths per 100,000 Population by Race/Ethnicity [Internet]. State Health Facts. [cited 2015 Dec 21]. Available from: http://kff.org/other/state-indicator/death-rate-by-raceethnicity/

3. Poslusna K, Ruprich J, Vries JHM de, Jakubikova M, Van&#39 P, Veer T. Misreporting of energy and micronutrient intake estimated by food records and 24 hour recalls, control and adjustment methods in practice. Br J Nutr. 2009 Jul;101(S2):S73–85.

4. Basu S, Seligman HK, Gardner C, Bhattacharya J. Ending SNAP Subsidies For Sugar-Sweetened Beverages Could Reduce Obesity And Type 2 Diabetes. Health Aff (Millwood). 2014 Jun 1;33(6):1032–9.

5. Deaton A, Muellbauer J. An almost ideal demand system. Am Econ Rev. 1980;312–26.

6. Andreyeva T, Long MW, Brownell KD. The Impact of Food Prices on Consumption: A Systematic Review of Research on the Price Elasticity of Demand for Food. Am J Public Health. 2010 Feb;100(2):216–22.

7. Hall KD, Sacks G, Chandramohan D, Chow CC, Wang YC, Gortmaker SL, et al. Quantification of the effect of energy imbalance on bodyweight. Lancet. 2011 Aug 27;378(9793):826–37.

8. Troiano RP, Berrigan D, Dodd KW, MâSse LC, Tilert T, Mcdowell M. Physical Activity in the United States Measured by Accelerometer: Med Sci Sports Exerc. 2008 Jan;40(1):181–8.

9. Patel AV, Hildebrand JS, Gapstur SM. Body Mass Index and All-Cause Mortality in a Large Prospective Cohort of White and Black U.S. Adults. PLoS ONE. 2014 Oct 8;9(10):e109153.

10. Flegal KM, Kit BK, Orpana H, Graubard BI. Association of all-cause mortality with overweight and obesity using standard body mass index categories: A systematic review and meta-analysis. JAMA. 2013 Jan 2;309(1):71–82.

11. Durazo-Arvizu RA, McGee DL, Cooper RS, Liao Y, Luke A. Mortality and Optimal Body Mass Index in a Sample of the US Population. Am J Epidemiol. 1998 Apr 15;147(8):739–49.

12. Calle EE, Thun MJ, Petrelli JM, Rodriguez C, Heath CW. Body-Mass Index and Mortality in a Prospective Cohort of U.S. Adults. N Engl J Med. 1999 Oct 7;341(15):1097–105.

13. Kivimäki M, Ferrie JE, Batty GD, Smith GD, Elovainio M, Marmot MG, et al. Optimal Form of Operationalizing BMI in Relation to All-cause and Cause-specific Mortality: The Original Whitehall Study. Obesity. 2008;16(8):1926–32.

14. Adams KF, Schatzkin A, Harris TB, Kipnis V, Mouw T, Ballard-Barbash R, et al. Overweight, obesity, and mortality in a large prospective cohort of persons 50 to 71 years old. N Engl J Med. 2006 Aug 24;355(8):763–78.

15. Park S-Y, Wilkens LR, Murphy SP, Monroe KR, Henderson BE, Kolonel LN. Body mass index and mortality in an ethnically diverse population: the Multiethnic Cohort Study. Eur J Epidemiol. 2012 Jul;27(7):489–97.
